# The interplay between hormonal vitamin D and lipopolysaccharide signaling on human neutrophil transcriptional responses

**DOI:** 10.3389/fimmu.2025.1683913

**Published:** 2025-10-03

**Authors:** Aiten Ismailova, Reyhaneh Salehi-Tabar, Nailya Ismailova, Olivia Dumas, James Saliba, Volker Blank, John H. White

**Affiliations:** ^1^ Department of Physiology, McGill University, Montreal, QC, Canada; ^2^ Department of Microbiology and Immunology, McGill University, Montreal, QC, Canada; ^3^ Department of Medicine, McGill University, Montreal, QC, Canada

**Keywords:** vitamin D, lipopolysaccharide, neutrophils, transcriptional responses, innate immunity, antibacterial, cathelicidin antimicrobial peptide, CYP24A1

## Abstract

**Introduction:**

Biologically active vitamin D (1,25-dihydroxyvitamin D or 1,25D) has emerged as a key regulator of human innate immunity. 1,25D signaling in macrophages strongly induces the expression of neutrophil chemoattractants, such as IL-8/CXCL8. Meta-analysis of vitamin D-regulated expression profiles has suggested that 1,25D may regulate granule formation in granulocytic cells. Here, we have examined the effects of 1,25D signaling on human neutrophil gene expression, alone and in combination with the inflammatory signal lipopolysaccharide (LPS). These studies are of interest because, whereas 1,25D signaling boosts innate immunity, it is anti-inflammatory.

**Methods and results:**

We determined the effects of 1,25D alone and in combination with LPS on gene expression of primary human neutrophils by RNAseq. LPS did not affect or slightly enhanced the expression of several well-characterized 1,25D-target genes, but strongly suppressed that encoding the 1,25D catabolic enzyme CYP24A1. Chromatin immunoprecipitation (ChIP) assays revealed that 1,25D-dependent vitamin D receptor (VDR) binding to the major *CYP24A1* enhancer was eliminated in neutrophils treated with LPS, whereas binding to other 1,25D-target genes was unaffected. Notably, LPS induced binding of transcriptional repressors MAFF and BACH1 to the major *CYP24A1* enhancer region. In other studies, pathway analyses revealed that 1,25D suppressed LPS-induced genes encoding inflammatory proteins. In addition, RNAseq and confirmatory RT/qPCR studies revealed that 1,25D, both on its own and in combination with LPS, increased mRNA expression of genes encoding antimicrobial components of secretory granules, including that encoding cathelicidin antimicrobial peptide (CAMP). Consistently, exposure of neutrophils to 1,25D enhanced bacterial killing, as revealed by a 20-25% reduction in *E. coli* colonies incubated with 1,25D-treated neutrophil conditioned media. The increased bacterial killing by 1,25D is mediated by 1,25D-induced secretion of cathelicidin, as an antibody against LL-37, the active form of cathelicidin, blocked antimicrobial activity.

**Discussion:**

Collectively, the data suggest that LPS prolongs vitamin D signaling by suppressing expression of the 1,25D catabolic enzyme CYP24A1. 1,25D signaling in the presence of LPS attenuates the expression of several genes associated with LPS inflammatory responses, whereas 1,25D in the absence or presence of LPS enhances the release of antibacterial proteins secreted by neutrophils in response to infection.

## Introduction

Initially recognized for its critical role in calcium and phosphate homeostasis, the hormonal form of vitamin D (1,25(OH)2D3 or 1,25D) is now understood to have multiple physiological effects, including a role in boosting innate immunity (1–3). While one can acquire vitamin D through dietary intake, supplements, or exposure to sufficient UVB radiation, diets lacking in vitamin D, avoidance of sunlight, and wearing conservative clothing contribute to a widespread deficiency. Clinical studies have shown that people who are deficient in vitamin D have increased risks of bacterial infections (2). Vitamin D deficiency is linked with an increased risk of dental caries (4). Moreover, vitamin D supplementation has been shown to decrease the severity and frequency of relapse in patients with Crohn’s disease, an inflammatory bowel disease characterized by defective intestinal innate immunity (5–8). It is therefore important to fully understand the molecular mechanisms of vitamin D signalling in innate immune responses to pathogen threat.

The activation of vitamin D occurs primarily in the liver by 25-hydroxylation, followed by 1α-hydroxylation, catalyzed by CYP27B1 in peripheral tissues, including cells of the immune system (9, 10). This results in the formation of the active form 1,25-dihydroxyvitamin D (11), which activates the vitamin D receptor (VDR). The VDR is a nuclear receptor that regulates gene expression by binding to specific regions on DNA called vitamin D response elements (VDREs), direct repeats of PuGG/TTCA separated by 3 base pairs (12). The 1,25D-bound VDR can directly and indirectly regulate the expression of more than 1000 genes (13) in a tissue- and species-specific manner (14).

Consistent with the pleiotropic actions of vitamin D, the VDR and CYP27B1 are widely expressed in tissues unrelated to calcium homeostasis, such as activated macrophages and dendritic cells (11, 15), implying local 1,25D production in cells. In activated macrophages and dendritic cells, production of CYP27B1 is controlled by immune signals such as interferon γ (IFN-γ), a cytokine released by pro-inflammatory Th1 cells, and bacterial lipopolysaccharide (LPS). LPS signals through toll-like receptor 4 (TLR4), a member of a large family of so-called pattern recognition receptors (PRRs) (16). Moreover, there is evidence that 1,25D can directly contribute to the host innate immune response by activating transcription of a number of genes, including those encoding antibacterial peptides (AMPs), PRRs, regulators of autophagy and cytokines in human monocytes, neutrophils and epithelial cells (1). Of note, 1,25D robustly induces the expression of *CAMP* (encoding cathelicidin antimicrobial peptide), whose active form (LL-37) exhibits potent anti-bacterial and anti-viral activity (17). 1,25D signaling also suppresses intracellular growth of *M. tuberculosis* and robustly enhances infection-induced interleukin (IL)-1β production in human macrophages (18).

Preclinical studies on the effect of 1,25D on innate immune transcriptional responses have been mostly carried out in monocytic or epithelial cells, whereas limited studies have been performed in granulocytic cells such as neutrophils. Neutrophils make up the largest portion of the granulocyte population (19), and individuals with congenital neutrophil deficiencies often suffer from serious infections (20, 21), highlighting the crucial role of these cells in immune defense. Neutrophils employ various techniques to eliminate microbes, including engulfing them (phagocytosis), releasing stored substances (degranulation), generating reactive oxygen species (respiratory burst), and deploying neutrophil extracellular traps (NETs) (19, 22). Neutrophils are not as transcriptionally silent as was once previously considered (23) and are instead now known to be transcriptionally active, which directly affects their functions (e.g. phagocytosis, bactericidal activity, apoptosis) (24–34). Within the past 20 years, there has been more effort directed toward uncovering transcriptional events in neutrophils following infection or inflammation, and systems biology-level approaches have provided significant insight into the role of these cells during host-pathogen interactions (26, 32, 34).

Our interest in these cells stemmed from our recent large-scale vitamin D-regulated gene expression re-analysis of 94 expression profiles, which suggested that 1,25D may regulate granule formation (35). However, these studies were performed in undifferentiated human HL60 promyelocytic leukemia cells, which represent a poor model for differentiated neutrophils. Moreover, we previously found that 1,25D stimulated expression and secretion of IL-8/CXCL8 in *Mtb*-infected macrophages (18). Given that IL-8 attracts neutrophils to sites of inflammation and/or infection (36), we wondered what the effect of 1,25D was on neutrophil transcriptomic responses. These studies are of particular interest because, while 1,25D signaling boosts innate immunity, it is also anti-inflammatory. Therefore, we carried out gene expression profiling studies in primary human neutrophils treated with 1,25D and/or LPS as an inflammatory signal. Interestingly, we found that LPS strongly but selectively repressed the 1,25D-induced expression of *CYP24A1*, which encodes the enzyme that initiates 1,25D catabolism. This occurred through the induced binding of transcriptional repressors MAFF and BACH1 to a VDRE downstream of the *CYP24A1* gene. In addition, 1,25D substantially altered the strong transcriptional responses of neutrophils to LPS. 1,25D, alone and in combination with LPS, regulated various neutrophil innate immune functions, including interleukin signaling and degranulation. Further, it suppressed the expression of genes encoding LPS-induced inflammatory cytokines. In conclusion, these data shed new light on an additional mechanism by which vitamin D signaling regulates the innate immune system.

## Results

### Exposure to LPS alters the transcriptional responses of neutrophils to 1,25D

**Figure 1:**
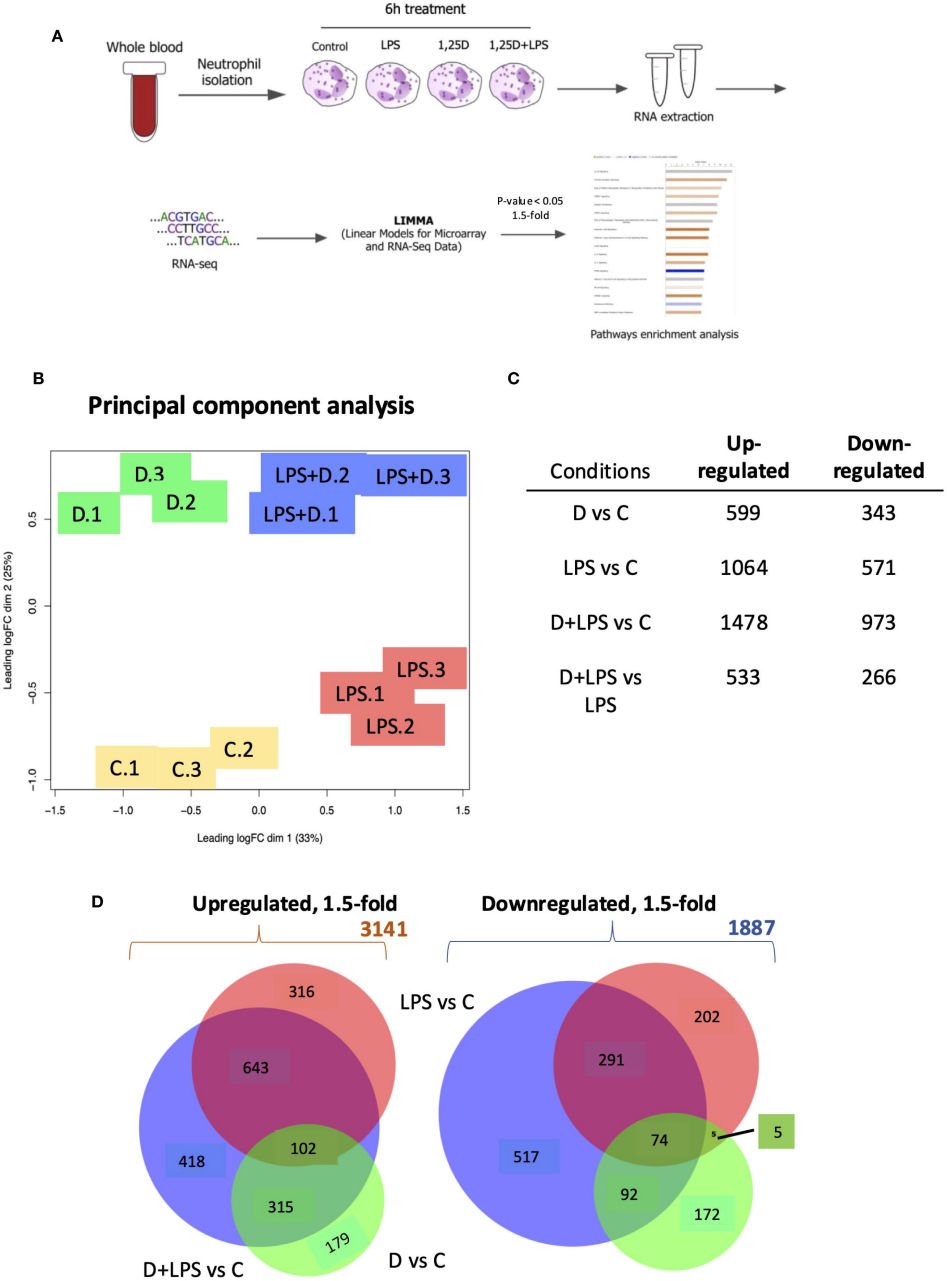
RNAseq analysis of primary human neutrophils treated in the presence or absence of 1,25D and in combination with LPS. **(A)** Schematic of neutrophil RNAseq experiment. **(B)** Principal component analysis of triplicate isolates of primary human neutrophils treated with control **(C)**, 1,25D **(D)**, LPS and 1,25D in combination with LPS (LPS+D). **(C)** Table of differentially expressed genes regulated 1.5-fold (p<0.05) by 1,25D and LPS alone relative to control as well as by 1,25D in combination with LPS relative to control. **(D)** Venn diagrams of 1.5-fold regulated gene expression changes in primary human neutrophils treated by 1,25D (green) and LPS (red) alone, as well as by 1,25D in combination with LPS relative to C (blue).

As neutrophils are among the first leukocytes recruited to sites of inflammation and infection, we investigated how 1,25D regulated their transcriptional responses. Neutrophils express the vitamin D receptor (VDR), and our preliminary studies (not shown) and published data showed that 1,25D upregulates known target genes such as those encoding the co-receptor for toll-like receptors *CD14* (37), *CYP24A1* and *CAMP* (38). To test for the effects of 1,25D on neutrophil gene expression in the presence of an inflammatory signal, we treated primary cultures of human neutrophils for 6h with 1,25D and LPS alone or in combination. Control experiments showed that these treatments did not affect neutrophil viability, as tested by flow cytometry (**Supplementary Figure S1**). We probed the relationship between LPS and 1,25D signaling in primary human neutrophils by performing RNAseq analysis (**Supplementary File S1**). Three isolates of primary cells were stimulated with 100 nM of 1,25D alone or in combination with 100 ng/ml LPS or treated with vehicle for 6h (**Figure 1A**). 100 ng/ml of LPS was employed in order to mimic systemic inflammation; this dose was shown to induce maximal gene expression of cytokines and other signature LPS-regulated genes (39), and has been used in other publications (40–44).

The results confirmed the robust expression of the *VDR* under all conditions (**Supplementary Table S1**). Principal component analysis revealed that each treatment condition produced distinct expression profiles and that those of each of the triplicates were highly concordant (**Figure 1B**). LPS- and 1,25D-regulated gene expression profiles were largely distinct. ~900 differentially expressed genes (DEGs) were significantly up- or downregulated by 1,25D at least 1.5-fold, and a further 900 DEGs were regulated by 1,25D in combination with LPS compared to LPS or 1,25D alone (**Figure 1C**). Venn diagram depiction of the data revealed that the effect of 1,25D was more modest on its own than that of LPS (**Figure 1D**). Conversely, gene expression regulated by LPS and 1,25D together had a greater overlap with genes regulated by LPS alone (**Figure 1D**). While 1,25D affected the magnitude of transcriptional responses to LPS, it rarely reversed LPS-regulated gene repression (**Supplementary Figure S3**).

**Figure 2:**
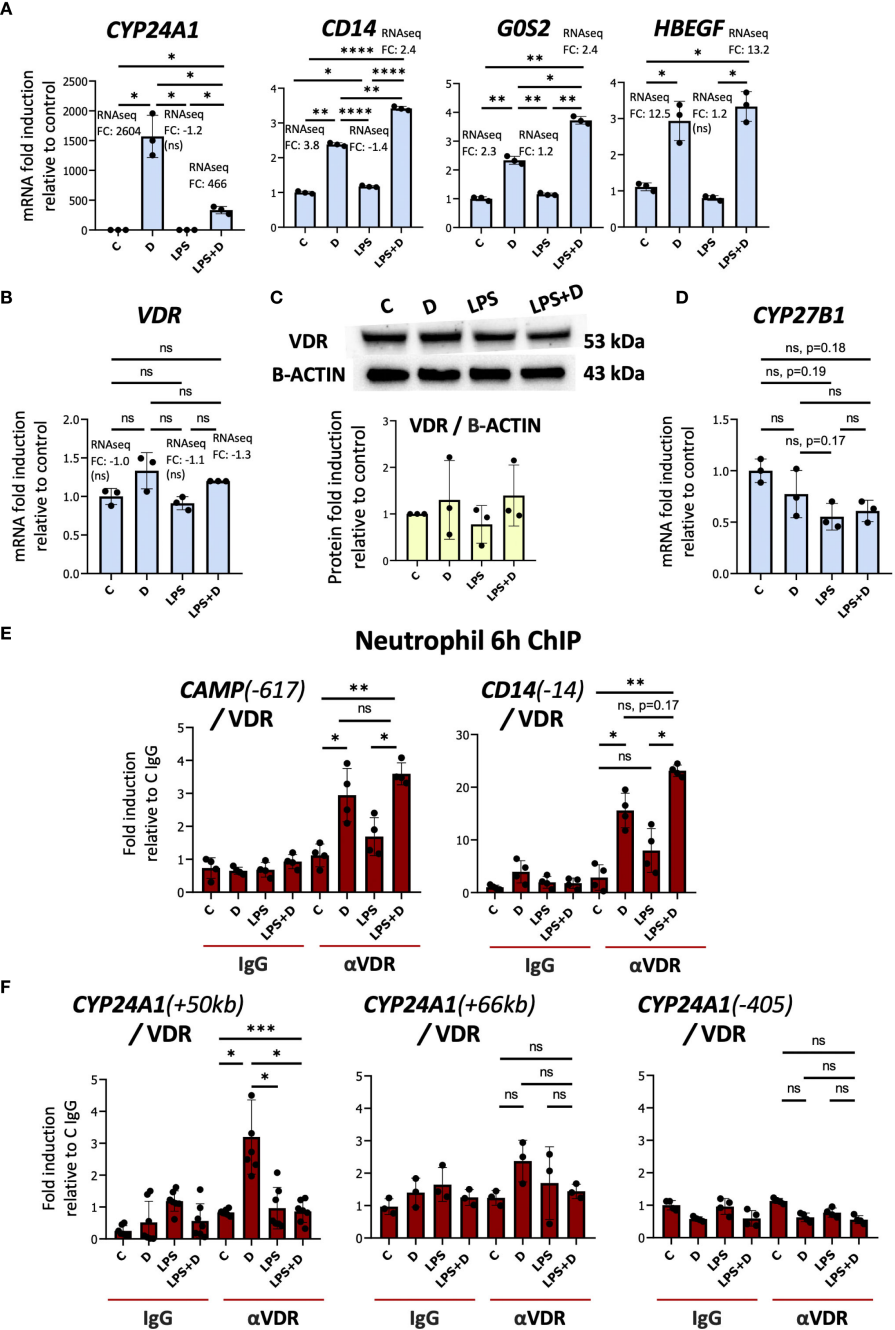
The transcriptional responses of neutrophils to LPS and hormonal vitamin D alone and in combination with each other. **(A)** Primary human neutrophils are responsive to 1,25D **(D)** as demonstrated by mRNA induction of *CYP24A1, CD14, G0S2*, and *HBEGF*. **(B)** RT-qPCR analysis of VDR gene expression by 1,25D and LPS alone or in combination. **(C)** Western blot of VDR in neutrophils and quantification relative to Beta-ACTIN. Graphics mean ± SD from 3 biological replicates and unpaired, one-way ANOVAs followed by Tukey’s *post hoc* test for multiple comparisons were used (ns ≥0.05). **(D)** RT/qPCR analysis of *CYP27B1* gene expression in neutrophils treated with or without LPS. RT/qPCR graphics are representative of 2 or 3 biological replicates. Graphics mean ± SD from 3 technical replicates from a representative sample, and paired, one-way ANOVAs followed by Tukey’s *post hoc* test for multiple comparisons were used (*P ≤ 0.05). **(E, F)** Analysis of the association of VDR with up- and downstream regulatory regions of *CAMP, CD14* **(E)** and *CYP24A1* **(F)** (based on hg38 genome assembly) by ChIP assay in neutrophils treated with or without 1,25D in the presence or absence of LPS. ChIPs are representative of 3–4 biological replicates. Graphics are mean ± SD from at least 3 technical replicates from a biological sample, and paired one-way ANOVAs followed by Tukey’s *post hoc* test for multiple comparisons were used (*P ≤ 0.05, **P ≤ 0.01, ***P ≤ 0.001, ****P ≤ 0.0001 and ns ≥0.05). ChIP values are normalized to input for each condition and expressed as a fold enrichment relative to IgG control.

Notably, LPS treatment strongly suppressed 1,25D-induced expression of *CYP24A1*, whereas it tended to boost the effect of 1,25D on other VDR target genes (**Figure 2A**). Decreased *CYP24A1* expression was also reported in 1,25D-treated macrophages infected with virulent and non-virulent strains of *Mtb* (18). LPS had no significant effect on the expression of VDR mRNA or protein (**Figures 2B, C**, **Supplementary Table S1**). In addition, the gene counts for *CYP27B1*, encoding the 1α-hydroxylase, were very low and were suppressed by LPS (**Figure 2D**, **Supplementary Table S1**). This observation contrasts with the stimulatory effect of LPS/TLR4 signaling on *CYP27B1* expression in macrophages (45), and indicates that LPS does not stimulate endogenous production of 1,25D in neutrophils. In addition, there is a non-specific trend towards inhibition by 1,25D of *CYP27B1* expression, which is not observed in macrophage-like cells (18) but is reminiscent of the repressive effect of 1,25D on *CYP27B1* in the kidney (46).

### 1,25D-dependent VDR binding to the +50kb *CYP24A1* enhancer is suppressed in the presence of LPS

To address the mechanisms underlying LPS-regulated suppression of *CYP24A1* expression, we performed ChIP assays of the VDR in multiple isolates of primary human neutrophils treated with vehicle, 1,25D, LPS, or LPS + 1,25D. Consistent with its effects on their genes, LPS maintained or slightly enhanced 1,25D-induced VDR binding to VDREs in the *CAMP* and *CD14* genes (**Figure 2E**, **Supplementary Figure S4**, **Supplementary Table S2**) (38, 47–51). ChIP on chip and ChIPseq studies have identified three principal VDR binding sites in the *CYP24A1* regulatory region, one promoter-proximal site and two downstream enhancers at +50 and +66 kb (52). We observed substantial 1,25D-dependent VDR binding to the +50kb site but not to the promoter-proximal nor the +66kb enhancer (**Figure 2F**, **Supplementary Figure S5**). Remarkably, 1,25D-dependent VDR binding to the +50kb enhancer was eliminated in neutrophils treated for 6h with LPS (**Figure 2F**, **Supplementary Figure S5A**). Interestingly, chromosome conformation capture assay in human colonic LS180 cells showed that the +50kb site is located structurally immediately adjacent to the *CYP24A1* promoter (52). The +50kb site has also been identified in VDR ChIPseq studies of 1,25D-treated undifferentiated and phorbol 12-myristate 13-acetate (PMA)-differentiated monocytic THP-1 cells (25, 26).

**Figure 3:**
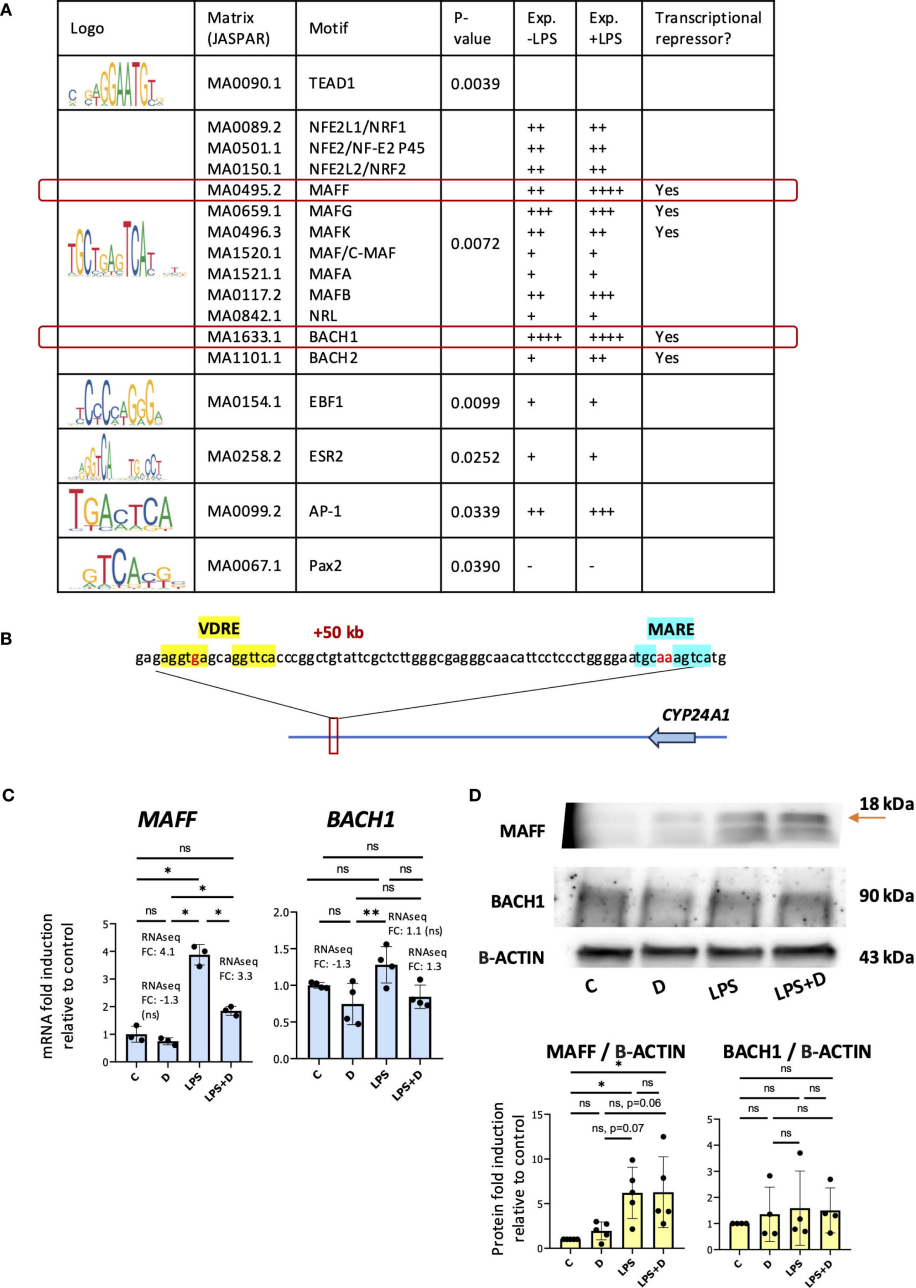
MAF recognition element enriched at the *CYP24A1* + 50kb downstream enhancer. **(A)** Table of enriched transcription factor motifs at the *CYP24A1* + 50kb enhancer. Exp. = expression level by RNAseq gene counts. **(B)** Schematic diagram of the human *CYP24A1* locus displayed with the +50kb position of the VDRE (highlighted in yellow) and MAF recognition element (MARE) (in blue font) with nucleotide bases indicated on chromosome 20 (hg19). In red font are the nucleotide mismatches. **(C)** RT/qPCR analysis of *MAFF* and *BACH1* gene expression in neutrophils treated with or without 1,25D and in the presence or absence of LPS. Graphics are representative of 3 biological replicates. Graphics mean ± SD from 3 technical replicates from a representative sample and paired, one-way ANOVAs followed by Tukey’s *post hoc* test for multiple comparisons were used (*P ≤ 0.05, **P ≤ 0.01 and ns ≥0.05). **(D)** Western blot analysis of MAFF and BACH1 and quantification relative to Beta-ACTIN. Graphics mean ± SD from 3–5 biological replicates and unpaired, one-way ANOVAs followed by Tukey’s *post hoc* test for multiple comparisons were used (*P ≤ 0.05 and ns ≥0.05).

Given that LPS treatment had no effect on VDR protein expression, we hypothesized that LPS signaling induced a repressive transcriptional environment in the region of the +50kb enhancer. To assess this, we inputted the *CYP24A1* + 50 kb enhancer region sequence from the hg38 genome into the Transcription Factor Affinity Prediction (TRAP) web tool (http://trap.molgen.mpg.de/cgi-bin/home.cgi) (see Methods and Materials for details) to search for transcription factor motifs. Among other motifs, this identified the “TGCTGAGTCA” sequence, also known as the MAF recognition element (MARE) and the cap’n’collar (CNC)-small MAF (sMAF)-binding element, a consensus binding site of MAF family proteins. This family is composed of small MAF oncoproteins (MAFF, MAFG, and MAFK) and large MAF proteins (C-MAF, MAFA, MAFB and NRL), and the cap’n’collar (CNC) family of transcription factors (NFE2 (NF-E2 p45), NFE2L1 (NRF1), NFE2L2 (NRF2), BACH1 and BACH2) (53) (**Figure 3A**). The near-consensus “TGCTGAGTCA” motif is located 48 bp upstream of the +50kb VDRE (**Figure 3B**). Large MAF homodimers and heterodimers of small MAF and CNC family of transcription factors are MARE-dependent activators, whereas heterodimers of small MAF with either BACH1 or BACH2 are MARE-dependent repressors (53–59). Interestingly, relative to the other enriched TFs, *BACH1* and *MAFF* were the most highly expressed and *MAFF* expression was induced by LPS at the gene expression level in our RNAseq analysis of neutrophils (see below) (**Figure 3A**, **Supplementary Table S1**) (39). Previous studies revealed the importance of MAFF in inflammatory responses (60–63). BACH1 is involved in the suppression of anti-inflammatory M2 macrophage differentiation (64, 65) as well as in the induction of inflammation observed in atherosclerosis (66) and rheumatoid arthritis (67). We could not find enriched MARE at the other *CYP24A1* -405b and +66kb enhancers. We confirmed increased gene and protein expression of MAFF in LPS-stimulated neutrophils by RT/qPCR and Western blot analyses (**Figures 3C, D**). The major MAFF band is at 18 kDa, and other smaller/minor bands represent other isoforms with a similar pattern to another study (68). BACH1 mRNA and protein were constitutively expressed and were not induced by LPS (**Figures 3C, D**).

### Elevated binding of transcriptional repressors MAFF and BACH1 is induced by LPS at the *CYP24A1* + 50kb enhancer

**Figure 4:**
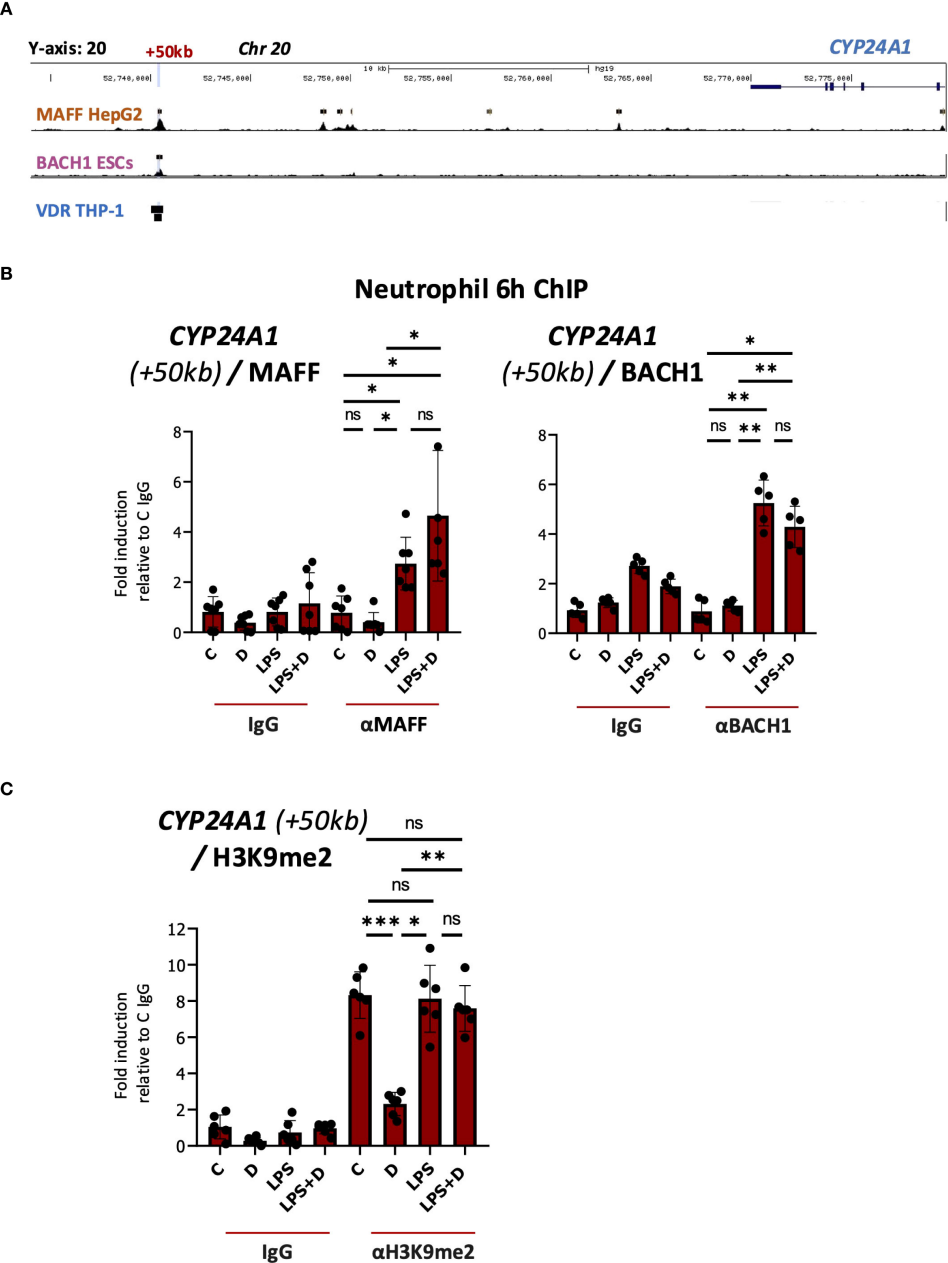
Enhanced binding of MAFF to the *CYP24A1* downstream enhancer as assessed by ChIP assay in LPS and LPS + 1,25D-treated neutrophils. **(A)** UCSC browser image showing VDR, MAFF and BACH1 ChIPseq tracks at the *CYP24A1* locus. The +50kb site of VDR, MAFF and BACH1 binding is highlighted in blue. The black boxes indicate bona fide peaks as determined by the corresponding ChIPseq studies. **(B)** Analysis of the association of MAFF, BACH1 and **(C)** H3K9me2 with the +50kb downstream enhancer of *CYP24A1* (based on hg38 genome assembly) by ChIP assay in neutrophils treated with or without 1,25D in the presence or absence of LPS for 6h. Graphics are representative of at least 3 biological replicates. Graphics mean ± SD from 5–6 technical replicates from a representative sample and paired, one-way ANOVAs followed by Tukey’s *post hoc* test for multiple comparisons were used (*P ≤ 0.05, **P ≤ 0.01, ***P ≤ 0.001 and ns ≥0.05). ChIP values are normalized to input for each condition and expressed as a fold relative to control IP.

We performed a series of *in silico* and directed ChIP experiments to determine if MAFF bound to the MARE in the +50kb enhancer. Importantly, we found a MAFF ChIPseq peak identified previously in hepatocyte HepG2 cells (**Supplementary Table S2**) that corresponds to the MARE in the *CYP24A1* + 50kb region (**Figure 4A**). We also found evidence for BACH1 binding to the +50kb region in a ChIPseq dataset from embryonic stem cells (**Figure 4A**, **Supplementary Table S2**). To determine whether MAFF and BACH1 interact with the *CYP24A1* enhancer, we performed ChIP assays in several isolates of vehicle-, 1,25D-, LPS- and LPS + 1,25D-treated neutrophils. Importantly, LPS induced MAFF and BACH1 binding to the *CYP24A1* + 50kb downstream regulatory region in the absence or presence of 1,25D (**Figure 4B**, **Supplementary Figures S6A, B**). BACH1 was shown to maintain the state of suppressive dimethyl acetylated histone 3 marker (H3K9me2) (69). Further, the same study found that overexpression of BACH1 resulted in decreased chromatin accessibility but increased binding of H3K9me2 at the promoters of target genes in human aortic smooth muscle cells (HASMCs). Moreover, the ChIPseq signal profile of H3K9me2 is greater at BACH1 enriched regions in BACH1 overexpressed HASMCs than control HASMCs (69). Accordingly, LPS increased binding of H3K9me2 to the *CYP24A1* + 50kb enhancer in the absence or presence of 1,25D (**Figure 4C**, **Supplementary Figure S6C**). In contrast, this histone mark was reduced with 1,25D treatment alone, thus providing more evidence for a repressive transcriptional environment induced with LPS treatment at the +50kb CYP24A1 enhancer.

### 1,25D regulates expression of several genes encoding components of secretory granules both on its own and in combination with LPS

**Figure 5:**
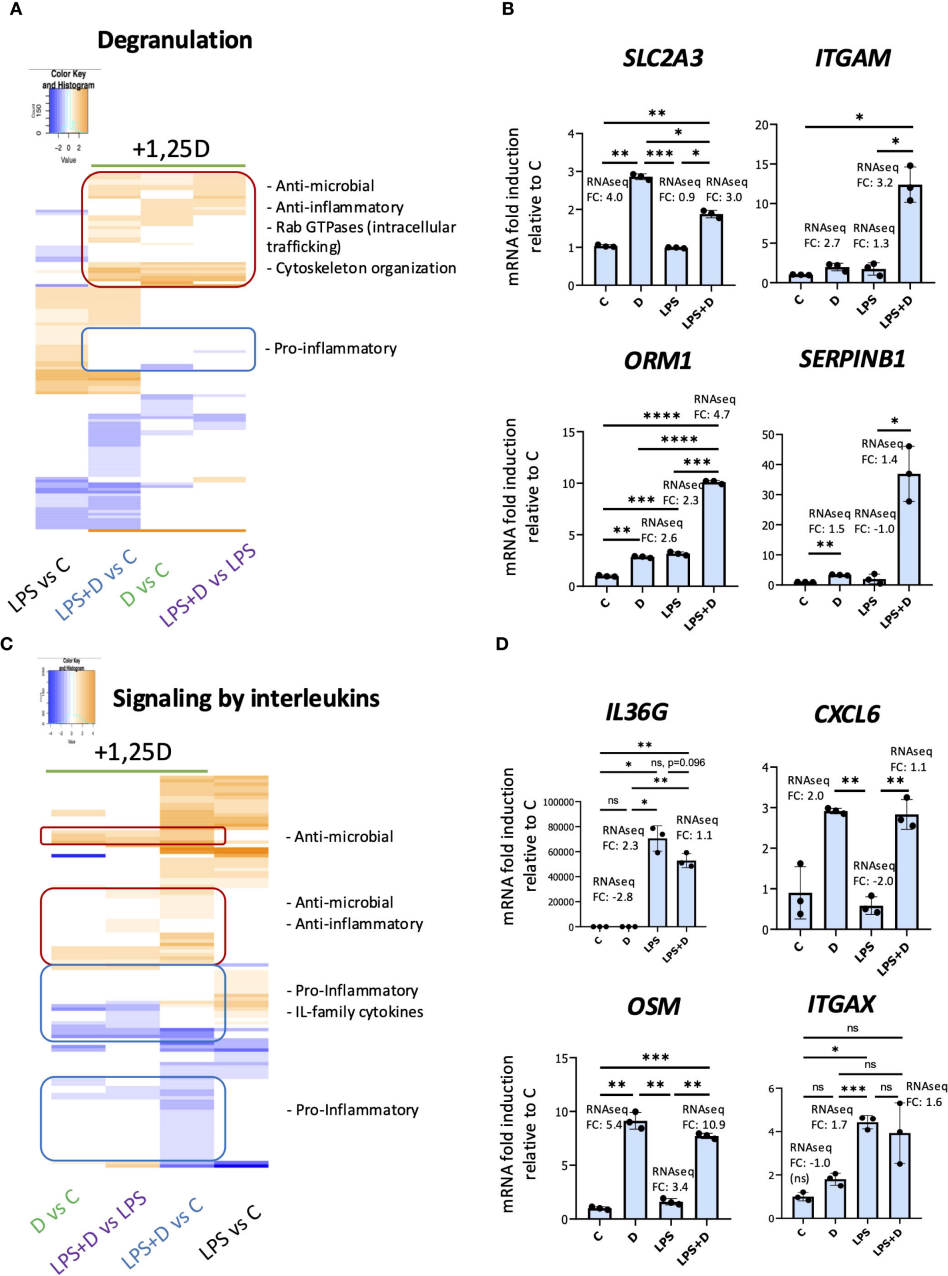
1,25D, in the presence and absence of LPS, may regulate degranulation and signaling by interleukins in primary human neutrophils. Heatmaps of DEGs within the degranulation **(A)** and signaling by interleukins **(C)** categories based on the Reactome pathway analysis. Note that hierarchical clustering was performed to generate the heatmaps, and as a result, the order of conditions differs between the two heatmaps. RT/qPCR validation of genes within the degranulation **(B)** and signaling by interleukin reactome categories **(D)**. Graphics are representative of 2 or 3 biological replicates. Graphics mean ± SD from 3 technical replicates from a representative sample and paired one-way ANOVAs followed by Tukey’s *post hoc* test for multiple comparisons were used (*P ≤ 0.05, **P ≤ 0.01, ***P ≤ 0.001, ****P ≤ 0.0001 and ns ≥0.05).

We performed pathways analyses to identify changes in gene expression signatures in the presence of 1,25D and/or LPS associated with neutrophil components and molecular signaling pathways (**Figure 5**, **Supplementary Figures S6**, **S7**, **Supplementary Files S2**, **S3**). 1,25D appeared to broadly suppress the effects of LPS on transcription of genes encoding cytokines and cytokine receptors (**Supplementary Figures S7A**, **8**). In addition, Gene Ontology representation analysis for biological processes revealed 1,25D-mediated regulation of several pathways important in neutrophil function, such as proliferation, adhesion and regulation of inflammatory responses (**Supplementary Figure S8B**, **Supplementary Figure S9**). Interestingly, gene ontology representation analysis for cellular components (**Supplementary Figure S7A**, **Supplementary File S2**) and reactome pathway analysis (**Supplementary Figure S7B**, **Supplementary File S3**), revealed regulated genes were associated with neutrophil granules, degranulation and interleukin signaling (**Figure 5**). Notably, 1,25D, in the presence or absence of LPS, regulates expression of genes encoding components of tertiary, specific and secretory granules (**Supplementary Figure S7A**, **Supplementary Table S3**; genes classified based on proteome profiling of human neutrophil granules (70)). To further investigate this, we produced heatmaps of DEGs within the granule and interleukin signaling categories (**Figures 5A, C**). From the degranulation heatmap (**Figure 5A**), 1,25D induced a subset of genes (enclosed in red) that are either not induced or repressed by LPS. These include genes encoding proteins involved in host innate immunity (e.g. *SLC2A3* and *SERPINB1*) (17, 71–83) and those with anti-inflammatory activity (e.g. *ORM1* and *ORM2*) (84–87). Further, the list of genes include Rab GTPases that traffic granules from the cytosol to the cell surface (e.g. *CRACR2A*) (88), genes encoding adhesion molecules (e.g. *ITGAM*) (89), and components of the cytoskeleton organization machinery (e.g. *DYNLT1*) (90), which, like the Rab GTPases, are important in degranulation (91–93). Conversely, 1,25D repressed a subset of genes that are upregulated by LPS alone (enclosed in blue), which, interestingly, included genes encoding pro-inflammatory proteins in neutrophil granules, such as prosaposin (*PSAP*), galectin-3 (*LGALS3*) (94) and plasminogen activator/urokinase (*PLAU*) (95, 96) (**Figure 5A**). We validated increased expression of some genes implicated in degranulation, such as *SLC2A3*, which encodes the GLUT3 glucose transporter (neutrophils rely on glycolysis for their effector functions) (97) and *ITGAM*, which encodes the membrane protein CD11b, a marker of secondary and tertiary granules (98–100) by RT/qPCR analysis in neutrophils (**Figure 5B**).

1,25D, in the presence or absence of LPS, regulated expression of genes encoding components of interleukin signaling (**Figures 5C, D**). 1,25D upregulated a subset of genes (enclosed in red) that are either not induced or repressed by LPS, and these included genes encoding anti-inflammatory proteins (e.g. *OSM* (101)) and those with antimicrobial activity (e.g. *LGALS9* (102–104), *CXCL6* (105, 106), *ITGAX* (107, 108)) (**Figures 5C, D**). However, another group of genes (enclosed in blue, top) are either not regulated or suppressed by 1,25D, but is upregulated by LPS. These included genes encoding pro-inflammatory and IL-family cytokines (e.g. *IL36G* (109) and *IL20* (110, 111)) (**Figures 5C, D**). In addition, 1,25D in the presence of LPS suppressed a cluster of genes encoding proinflammatory cytokines (e.g. *CCL19* (112, 113) and *CCL2* (114, 115)) that are otherwise not regulated by LPS (enclosed in blue, bottom, **Figure 5C**). This is in concordance with the notion that 1,25D is anti-inflammatory and that vitamin D sufficiency suppresses peripheral inflammatory immune responses.

**Figure 6:**
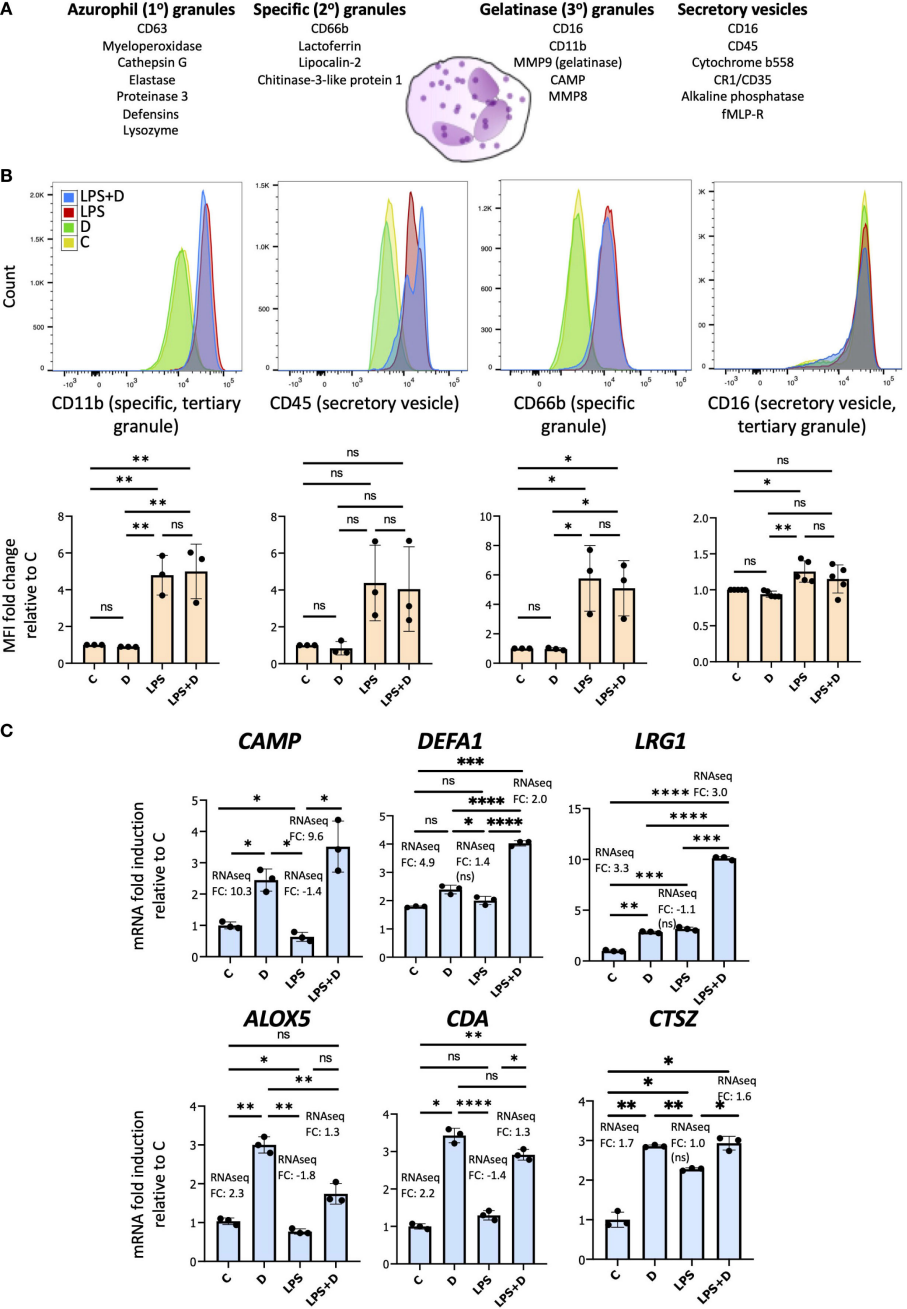
1,25D regulates gene expression of secreted neutrophil granule proteins in the absence and presence of LPS. **(A)** Schematic of the different granules contained within neutrophils and their membrane and secreted components. **(B)** Upper row: Representative flow cytometric analysis of cell surface markers of granules in neutrophils treated for 6h with 1,25D on its own and in combination with LPS. Representative fluorescence histograms of 3–5 biological replicates. Bottom row: Histograms of Mean fluorescence intensity (MFI) for 3–5 biological replicates. Statistical analyses: mean ± SD and unpaired, one-way ANOVAs followed by Tukey’s *post hoc* test for multiple comparisons were used (*P ≤ 0.05, **P ≤ 0.01 and ns ≥0.05). **(C)** RT-qPCR analysis of antimicrobial and anti-inflammatory secreted components of granules. Graphics are representative of 3 biological replicates. Graphics mean ± SD from 3 technical replicates from a representative sample and paired, one-way ANOVAs followed by Tukey’s *post hoc* test for multiple comparisons were used (*P ≤ 0.05, **P ≤ 0.01, ***P ≤ 0.001, ****P ≤ 0.0001 and ns ≥0.05).

Given that 1,25D alone or in combination with LPS regulates several genes whose products are implicated in degranulation, particularly in gelatinase (3°), specific (2°) and secretory granules, we investigated whether 1,25D can modulate degranulation by flow cytometric assessment of cell surface granule markers (100). There are four different types of granules within neutrophils, each one containing different antimicrobial proteins that are secreted upon inflammatory or pathogen challenge, as well as membrane proteins that are translocated to the cell surface during degranulation (**Figure 6A**) (116). However, 1,25D did not appear to regulate the delivery of cell surface markers of granules (**Figure 6B**). Nevertheless, our RNAseq suggests that 1,25D in the presence or absence of LPS may regulate the antimicrobial and anti-inflammatory secreted components of various granules (**Supplementary Table S3**). We confirmed the 1,25D-mediated induction of several genes encoding these proteins (*CAMP*, *DEFA1*, *LRG1*, *ALOX5*, *CDA* and *CTSZ*) by mRNA expression (**Figure 6C**).​ *CAMP* and *DEFA1* encode antimicrobial peptides (AMPs). Due to their amphipathic properties, both cathelicidin and defensin peptides disrupt bacterial membranes through interactions with hydrophobic and phospholipid components (17). Unlike *CAMP*, *DEFA1* appears to be uniquely regulated by 1,25D in the presence of LPS, and not by 1,25D alone (**Figure 6C**). In addition to AMPs, 1,25D, in the presence or absence of LPS, upregulated other host-defense implicated and anti-inflammatory genes such as: *LRG1*, which encodes a secreted glycoprotein containing leucine-rich repeats that serve as pattern recognition motifs for the innate immune system (76); *CTSZ* or cathepsin Z, which is a member of the family of antimicrobial and anti-inflammatory cathepsins or serine proteases (117, 118); *ALOX5*, an enzyme that enhances AMP production and pathogen killing by neutrophils (119), and *CDA*, a cytidine deaminase, which was shown to reduce viability of *E. Coli* (71). Unlike *LRG1*, gene expression of *ALOX5, CDA* and *CTSZ* does not appear to be further enhanced by 1,25D in the presence of LPS (**Figure 6C**). Overall, the data suggest that while 1,25D does not regulate the expression of cell surface markers of granules, it may regulate the secreted components of granules.

### 1,25D induces antibacterial activity against *E. coli* in neutrophils

**Figure 7:**
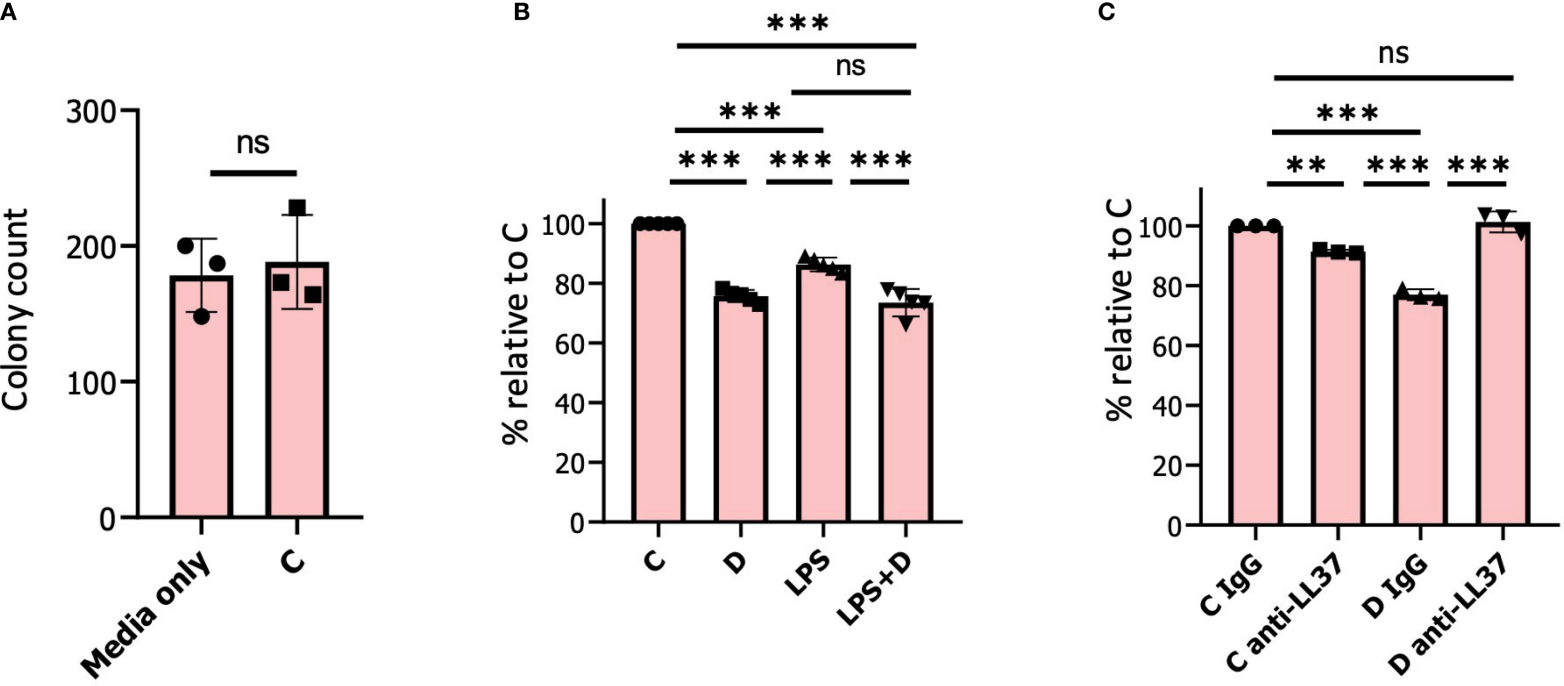
1, 25D induces antimicrobial activity in neutrophils. **(A)** 500 colony-forming units (CFU) of *E*. *Coli* were incubated in conditioned medium from neutrophils pre-treated for 6h with vehicle. Bacterial samples were incubated at 37 °C with shaking for 30 min prior to plating. The results are expressed as a percentage of bacterial colonies relative to media only incubated with *E*. *Coli*. **(B)** 500 CFU of *E*. *Coli* were incubated in conditioned medium from neutrophils pre-treated for 6h with either vehicle/control (C), 1,25D (D), LPS, and 1,25D+LPS (LPS+D). The results are expressed as a percentage of bacterial colonies relative to vehicle-treated neutrophils. **(C)** 500 CFU of *E*. *Coli* were incubated in conditioned medium from neutrophils pre-treated with vehicle or 1,25D for 6h. Prior to incubation with *E*. *Coli*, either IgG control or anti-LL-37 antibody was added to samples for 30 min. Graphics are mean ± SD from 3 or 5 biological replicates and unpaired, one-way ANOVAs followed by Tukey’s *post hoc* test for multiple comparison were used (**P ≤ 0.01, ***P ≤ 0.001 and ns ≥0.05).

A prediction from 1,25D inducing expression of genes encoding antimicrobial components of granules is that it may enhance neutrophil antibacterial activity. Therefore, to test for induction of antimicrobial activity, we conducted bacterial killing experiments using *E. coli* incubated with neutrophil-conditioned media (14, 38) (**Figure 7**). Consistent with our hypothesis, conditioned culture media from human neutrophils treated for 6h with 1,25D in the presence or absence of LPS modestly but significantly inhibited viability of *E. coli* (**Figure 7B**). There was no significant difference in bacterial killing using conditioned media of vehicle-treated neutrophils compared to media only (**Figure 7A**). Given that the *CAMP* gene is robustly induced by 1,25D in neutrophils, we were interested in determining the contribution of its encoding active peptide, LL-37, to secreted antibacterial activity. To this end, we used an anti-LL-37 antibody that blocked antimicrobial activity in lung airway surface liquid (120). Remarkably, incubation of conditioned media with this antibody, as opposed to control IgG, completely blocked 1,25D-induced antimicrobial activity (**Figure 7C**), confirming that secretion of LL-37 is the major component of 1,25D-enhanced antibacterial activity.

## Discussion

To date, analysis of innate immune regulation by vitamin D has been mostly conducted in monocytes and macrophages. However, neutrophils, the most abundant immune cells in the circulation, are also vital in host innate immunity and play an instrumental role in antimicrobial defense. In addition, neutrophil inflammatory responses must be regulated, as uncontrolled inflammation may be pathogenic; for instance, acute respiratory distress syndrome, associated with respiratory infections, is driven by overactive neutrophil inflammation (121). However, data on the effects of 1,25D signaling on neutrophil function remain limited. Our lab has previously demonstrated that 1,25D induced secretion of neutrophil chemokine IL-8 in macrophages infected with *Mtb*, implying enhanced recruitment of neutrophils to sites of infection (122). 1,25D may also influence granule formation in undifferentiated human promyelocytic leukemia HL-60 cells, as shown by our re-analysis of 94 human and mouse vitamin D-regulated expression profiles (35). Considering these data, we investigated the effect of 1,25D, in the presence or absence of the inflammatory signal LPS, on primary human neutrophil transcriptomic responses to determine whether the hormone may regulate neutrophil microbial activity.

RT/qPCR results suggested that *CYP27B1* expression in neutrophils was weak, and unlike monocytic cells, its expression was not induced by LPS. This was confirmed by our RNAseq gene counts and is consistent with a prior study (38). These results suggest that neutrophils do not generate 1,25D from circulating 25D in the presence of an inflammatory signal, but rather respond to 1,25D produced locally from macrophages at sites of infection or inflammation. Moreover, under the influence of LPS, 1,25D-induced *CYP24A1* expression was strongly repressed in neutrophils, suggesting that 1,25D catabolism is inhibited in LPS-treated neutrophils. Similar suppression of *CYP24A1* induction was previously observed in *Mtb*-infected macrophages (18), although the underlying mechanisms were not addressed. mRNA and protein expression of the VDR was not significantly affected by LPS, but ChIP assays of primary human neutrophils revealed decreased 1,25D-induced binding of the VDR in the presence of LPS to a previously identified enhancer 50 kb downstream of the *CYP24A1* TSS (52). Further ChIP studies showed that LPS induced binding of MAFF and BACH1, components of a transcriptional repressor complex, to the +50kb enhancer, to a near-consensus MARE adjacent to the VDRE. This is in line with a motif enrichment analysis of 1,25D-regulated genomic binding sites from ATACseq and RNAseq of THP1 cells, which revealed a shift from canonical VDR-RXR binding in 1,25D-treated cells to TGAGTCA-enriched motifs (MARE near-consensus sequence) in cells exposed to LPS alone or in combination with 1,25D (123). We also found that binding of the suppressive dimethyl acetylated histone 3 marker was induced with LPS in the absence or presence of 1,25D; this is indicative of transcriptional repression. Among the CNC family members, BACH1 and BACH2 heterodimerize with small MAF proteins to repress transcription (53). We found that, in comparison to other family members, *MAFF*, an inflammation-linked transcription factor (60), and *BACH1*, associated with repressing M2 anti-inflammatory macrophage differentiation (64, 65), were the most highly expressed in LPS-treated neutrophils. MAFF mRNA and protein expression were induced by LPS in primary human neutrophils, whereas BACH1 was constitutively expressed. Interestingly, increased MAFF and BACH1 heterodimeric binding at the MARE of the *LDLR* (which encodes the low-density lipoprotein receptor) promoter and ensuing suppression of *LDLR* expression were previously demonstrated in the presence of LPS in human Hep3B and murine AML12 liver cell lines (61). Moreover, similar to our study, MAFF induction by LPS was robust. However, LPS-mediated induction of BACH1 was modest (61), suggesting that LPS-induced expression of one of the heterodimeric partners is sufficient to drive increased binding of MAFF/BACH1 to the *CYP24A1* enhancer.

This study represents the first large-scale RNAseq study on neutrophils treated with 1,25D on its own and in combination with LPS to probe their individual or combined effects on neutrophil transcriptomic responses. Bioinformatic analysis revealed ~900 genes being regulated by 1,25D alone, and a further 900 genes regulated by 1,25D in combination with LPS. The majority of genes regulated by 1,25D and LPS alone and in combination were induced. This is contrary to transcriptomic analysis from human peripheral blood mononuclear cells (PBMCs), where single treatments of 1,25D and LPS diminished gene expression (124). Co-stimulation of LPS with 1,25D in PBMCs resulted in a reduced number of responsive genes (124), which is in contrast to the increased number of genes regulated by LPS+D in neutrophils compared to LPS or 1,25D alone. However, ATACseq and RNAseq from THP1 cells revealed that co-treatment with LPS and 1,25D altered chromatin accessibility at over 41,500 genomic regions and significantly regulated the expression of >2000 genes (123), which is in agreement with our data. Intriguingly, while 1,25D impacted the magnitude of LPS-mediated transcriptional responses, the hormone rarely reversed LPS-regulated gene repression in neutrophils. Pathway analyses suggested a role for 1,25D in the regulation of degranulation and signaling by interleukins. Degranulation was also noted as an enriched pathway in 1,25D-treated HL60 cells (35). Upon closer inspection, it appeared that 1,25D boosted expression of genes encoding anti-inflammatory and antimicrobial proteins within the degranulation network, while at the same time it inhibited LPS-induced expression of genes encoding pro-inflammatory mediators found in granules. This is consistent with previous reports of 1,25D as an anti-inflammatory and antimicrobial modulator of the immune system (1). Along the same vein, 1,25D broadly suppressed LPS-induced expression of genes encoding pro-inflammatory cytokines in our RNAseq, an observation also supported by smaller-scale studies in neutrophils (37, 125) and an RNAseq study from 1,25D and LPS-treated human PBMCs (124).

1,25D did not appear to regulate the delivery of surface markers of granules. However, we did provide evidence for 1,25D enhancing mRNA expression of genes encoding antimicrobial proteins found within granules, such as *CAMP*, *DEFA1*, *LRG1*, and *CTSZ*, which supports the notion that 1,25D regulates the secreted components of granules. A prediction of enhancing the expression of antimicrobial components of granules would be increased bacterial killing by 1,25D-treated cells. Indeed, we found that 1,25D treatment of neutrophils for 6 hours significantly increased secreted antimicrobial activity against *E. Coli*. Notably, this increase was abolished by incubation of conditioned media from neutrophils with an antibody against LL-37, the active peptide encoded by the *CAMP* gene. Granulocytic cells are a major source of circulating LL-37 due to their abundance and storage of LL-37 in granules released at sites of infection (126, 127). LL-37 is known to confer antibacterial activity against Gram-negative bacteria in *in vitro* experiments (128). Our lab has previously demonstrated that 1,25D robustly enhanced bacterial killing in epithelial cells treated with 1,25D for 24 and 48 hours (14, 38). Therefore, it is possible that the bactericidal effect of 1,25D in neutrophils may be even greater with longer incubation times. Due to the limited viability of primary human neutrophils cultured *in vitro* (6-8h) (129–133), we did not test extended periods of incubation. However, neutrophils *in vivo* may live 3 days or more (134–138), suggesting that 1,25D may act on neutrophils for a longer period.

In conclusion, we provide evidence of innate immune regulation by hormonal vitamin D in neutrophils. For the first time, we uncovered the mechanism of genomic regulation of LPS-mediated *CYP24A1* suppression in the presence of 1,25D via induced binding of transcriptional repressors MAFF and BACH1 to a *CYP24A1* enhancer. We show that in neutrophils, 1,25D suppresses inflammatory signals while at the same time it enhances anti-microbial activity, mainly by boosting expression of *CAMP*. These dual roles are key to the immunomodulatory effects of 1,25D. Future exploration in the physiological role of 1,25D on neutrophil function would shine more light on novel mechanisms of 1,25D-mediated regulation of immune responses to infection and inflammation.

## Materials and methods

### Human neutrophil isolation and treatment

Whole blood from consenting healthy donors was collected under McGill University Health Centre REB ethics # 23-03-044. Primary human neutrophils were isolated from blood using negative selection with the EasySep™ Direct Human Neutrophil Isolation Kit (STEMCELL) following the manufacturer’s instructions. Neutrophil purity was assessed by flow cytometry, measuring markers specific to various blood cell populations, including CD45 (for hematopoietic cells, excluding erythrocytes and platelets), CD16 (for natural killer cells, neutrophils, and macrophages), and CD66b (for granulocytes) (**Supplementary Figure S2**). Cell count was determined using an automatic cell counter (Bio-Rad), adjusting the concentration to between 5×10^5^ and 1×10^6^ cells/ml. Neutrophils were resuspended in tissue culture medium containing RPMI 1640 (1X with L-glutamine, sodium pyruvate, and 25mM HEPES, Wisent 350-006-CL), supplemented with 10% fetal bovine serum and penicillin/streptomycin (ScienCell 0503). The cells were then treated with 100nM 1,25D (BML-DM200, Enzo Life Sciences), 100ng/mL LPS (L3012-5MG, Sigma-Aldrich), or vehicle (dimethyl sulfoxide) for 6 hours. Annexin V/propidium iodide staining confirmed that after 6 hours, the neutrophils remained mostly viable (**Supplementary Figure S1**).

### RNA sequencing

RNA sequencing was conducted essentially as described (39). Briefly, total RNA was extracted from three isolates of 1,25D-, LPS-, 1,25D+LPS- and vehicle-treated neutrophils using the FavorPrep Blood/Cultured Cell Total RNA Mini Kit (FABRK 001, Favorgen) according to the manufacturer’s protocol. Biological replicates were generated from three independent neutrophil isolates from three different donors. Only RNA samples with an OD 260/280 ratio greater than 1.7 and an RNA integrity number (RIN) > 7 were retained for further analysis. These samples were then submitted to Génome Québec for paired-end sequencing with 100M reads on an Illumina NovaSeq PE100 sequencer. Library preparation was performed using the polyA Enriched RNA Library Preparation. All samples met quality standards as determined by QC reports from Genome Quebec and were included in the analysis. The quality of sequence reads was verified using FastQC, with poor-quality reads identified based on the Phred score, which is logarithmically related to base calling error probabilities. For all RNA-seq datasets, the Phred offset quality score exceeded 30, and the minimum fragment size for alignment was set to 50. Low-quality bases were trimmed from read ends using default settings in Trimmomatic, and quality was re-assessed using FastQC. Reads were mapped to the human GRCh38 genome assembly using HISAT2. Gene expression was quantified by counting uniquely mapped reads with StringTie, using default parameters. Normalization and differential gene expression analysis were conducted with the DESeq2 Bioconductor package. Genes with ≥|1.5| fold-change and adjusted p-values ≤ 0.05 were considered significant. The differentially expressed genes from the RNAseq analysis are provided in **Supplementary File S1**.

### Flow cytometry

Adherent neutrophils were detached by gently pipetting the tissue culture dishes up and down. Both adherent and suspension cells were then centrifuged at 500 rcf for 10 minutes and washed twice with ice-cold PBS. The supernatant was removed, and the cells were resuspended in FACS buffer (0.5-1% BSA in PBS) at a concentration of 1 × 10^6^ cells/mL. To block nonspecific binding, human FcR binding inhibitor (14-9161-73, eBioscience) was added. Neutrophil degranulation was determined by incubating the cells with 2 µg of anti-human PE-CD66b (392903, BioLegend), PE-CD16 (302007, BioLegend), Alexa Fluor 700-CD45 (368514, BioLegend), and APC-CD11b (301310, BioLegend) antibodies for 30 minutes at room temperature in the dark. Cell viability was assessed using the Vybrant Apoptosis Assay kit (V13242, Molecular Probes). After washing, cells were either cross-linked in 2% paraformaldehyde or immediately analyzed by flow cytometry for purity and viability, respectively. Flow cytometry acquisition was performed using a BD-LSRFortessa analyzer, monitoring at least 10,000 cells per sample. Data analysis was conducted using FlowJo software (TreeStar Inc.).

### Bioinformatics analysis

The overlap between 1,25D, LPS, and 1,25D+LPS is illustrated using Venn diagrams implemented by the *VennDiagram* package in R. Principal component analysis (PCA) was performed using the R function prcomp and visualized with the ggplot package. Enrichment analysis of Reactome pathways was performed using the ReactomePA package (139). Enrichment analysis of gene ontology representation analysis for biological processes and cellular components, as well as canonical pathways (Kyoto Encyclopedia of Genes and Genomes), was conducted using the clusterProfiler package (140). Heatmaps with hierarchical clustering were constructed using the heatmap.2 package in R. Peaks from VDR, MAFF, and BACH1 ChIPseq studies and datasets from the ENCODE consortium were aligned with the human genome (build hg19 or hg39) using the UCSC Genome Browser (http://genome.ucsc.edu/cgi-bin/hgGateway). To find sequence motifs enriched in the *CYP24A1* + 50kb enhancer, we extracted their sequence from the hg19 or hg38 genome and used this as input for the Transcription Factor Affinity Prediction (TRAP) web tool (http://trap.molgen.mpg.de/cgi-bin/home.cgi) using JASPAR vertebrates as the comparison library, human promoters as the control, and Benjamini-Hochberg as the correction (141). We used a p-value threshold of 0.05. This resulted in the enrichment of near-consensus motifs for MARE and CNC-sMaf binding elements.

### RNA extraction, reverse transcription and qPCR

RNA extraction was performed using the FavorPrep™ Tissue Total RNA Mini Kit (FATRK 001, Favorgen) according to the manufacturer’s instructions. Validation of RNAseq by RT/qPCR was conducted using neutrophil isolates from different donors than those used for the RNAseq. cDNA was synthesized from 100–500 ng of RNA using the 5× All-in-One RT Mastermix (G485, abm) and diluted 5 times. Quantitative polymerase chain reaction (qPCR) was conducted with BrightGreen 2×qPCR MasterMix (MasterMix-LR-XL, abm) on a Roche Applied Science LightCycler 96 machine. Gene expression was normalized to ZC2HC1C. All primers are listed in **Supplementary Table S4**.

### Western blotting and protein analysis

Western blotting and protein analysis were achieved as detailed (1). The antibody for MAFF was generously provided by Dr. Volker Blank and used at a dilution of 1:20,000. BACH1 (sc-271211, Santa Cruz, 1:100) and VDR (sc-13133, Santa Cruz, 1:500) primary antibodies were purchased from Santa Cruz. The anti-rabbit and anti-mouse IgG HRP-linked secondary antibodies were purchased from Cell Signaling Technology and used at recommended concentrations. We quantified changes in protein levels relative to control using Image Lab software after normalization to β-actin (#4970, Cell Signaling Technology, 1:500). Western blot images are representative of at least three biological replicates.

### Chromatin immunoprecipitation assays

ChIP assays were conducted as previously specified (39). The VDR antibody used for ChIP (4 µg/sample) is the same as for western blotting. IgG mouse antibody (sc-2025, Santa Cruz, 4 µg/sample) was purchased from Santa Cruz. MAFF (12771-1-AP, Proteintech) and BACH1 (14018-1-AP, Proteintech) ChIP antibodies were purchased from Proteintech and used at 2 µg/sample. H3K9me2 (#4658S, Cell Signaling Technology) and normal IgG rabbit antibodies (#2729S, Cell Signaling Technology) were purchased from New England Biolabs and were used at 2 µg/sample. Primer pairs used for ChIP assays are listed in **Supplementary Table S4**.

### Antimicrobial assays with neutrophil conditioned media

Antimicrobial assays were performed as previously described (14). *E. coli* was grown to early log phase at 37 °C in Luria–Bertani (LB) broth (800-060-LG, Wisent). 50 μl cultures in LB broth were diluted to 500 CFU with 150 μl of regular, non-conditioned medium as a negative control or conditioned medium from cells treated with 1,25D, LPS and LPS + 1,25D for 6h. Samples were incubated at 37 °C with shaking for 30 min, and bacteria were then plated onto LB agar (800-011-LG, Wisent) plates, and CFUs were counted after 18 h. The results for the conditioned medium experiments are expressed as a percentage of CFUs relative to bacteria cultured in non-conditioned medium. For the anti-LL-37 experiments, the conditioned media were treated with 1 µg/ml anti-LL-37 (HM2070, Hycult) or IgG (5415S, Cell Signalling Technology) for 30 min at 4 °C with shaking, before contacting *E. coli*.

### Statistics

A two-tailed t-test (Student’s t-test), conducted using GraphPad software, was used to assess the significance between two conditions. For four conditions, a one-way ANOVA followed by Tukey’s *post hoc* test for multiple comparisons was applied using GraphPad. A p-value of ≤ 0.05 was considered statistically significant. p-values were represented with the following symbols: **P ≤ 0.05, **P ≤ 0.01, ***P ≤ 0.001, ****P ≤ 0.0001, and ns ≥ 0.05. Results from RT/qPCR, western blotting, ChIP analyses, flow cytometry and antimicrobial assays are based on at least three biological replicates, and one-way ANOVAs were used to determine significance. Biological replicates refer to neutrophil isolates from different human blood donors, and technical replicates refer to repeated measurements of the same sample. Paired tests were used for technical replicates of a representative sample, while unpaired tests were used for biological replicates.

## Data Availability

The data presented in the study are deposited in the Gene expression omnibus (GEO) repository, accession number GSE308684.

## Supplementary material

Supplementary File S1 RNAseq of primary human neutrophils treated with and without 1,25D and in the absence or presence of LPS for 6h. Gene expression changes in 1,25D, LPS, LPS + 1,25D and vehicle-treated neutrophils identified by RNAseq. Fold change values, along with their corresponding p-values, are indicated respectively.

Supplementary File S2 Enriched pathways for Gene Ontology representation analysis for cellular processes of 1.5-regulated genes from 1,25D-, LPS- and LPS + 1,25D-treated neutrophils relative to control, as well as LPS + 1,25D-stimulated cells vs. LPS-treated.

Supplementary File S3 Enriched Reactome pathways of 1.5-regulated genes from 1,25D-, LPS- and LPS + 1,25D-treated neutrophils relative to control, as well as LPS + 1,25D-stimulated cells vs. LPS-treated.
