# Supplementary material for: The interplay between hormonal vitamin D and lipopolysaccharide signaling on human neutrophil transcriptional responses
Source: Front Immunol. 2025 Oct 3;16:1683913. doi: 10.3389/fimmu.2025.1683913 (PMC12531266; doi:10.3389/fimmu.2025.1683913)
Supplement: Supplementary File S1 — RNAseq of primary human neutrophils treated with and without 1,25D and in the absence or presence of LPS for 6h. Gene expression changes in 1,25D, LPS, LPS + 1,25D and vehicle-treated neutrophils identified by RNAseq. Fold change values, along with their corresponding p-values, are indicated respectively. [file DataSheet1.pdf]

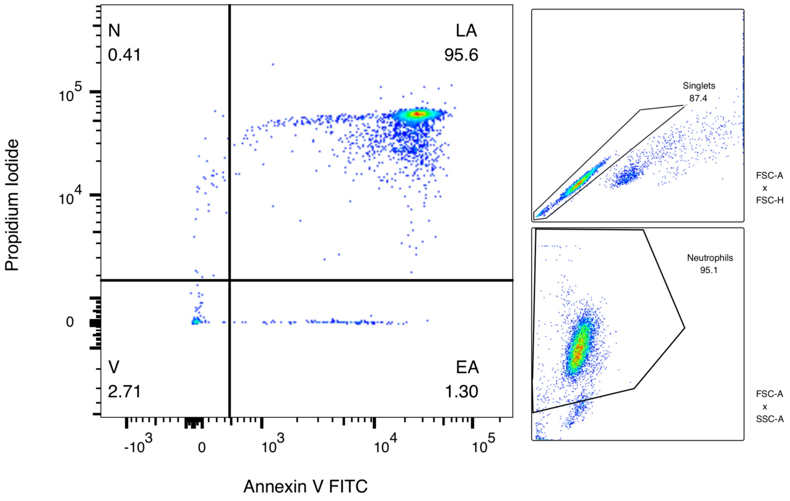

Positive control for apoptosis (neutrophils incubated at 62°C water bath, 20 minutes)

V = viable, N = necrotic, LA = late apoptotic, EA = early apoptotic

Control

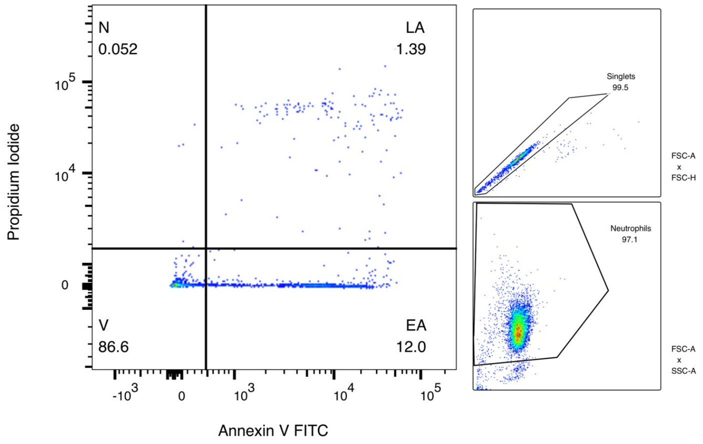

1,25D

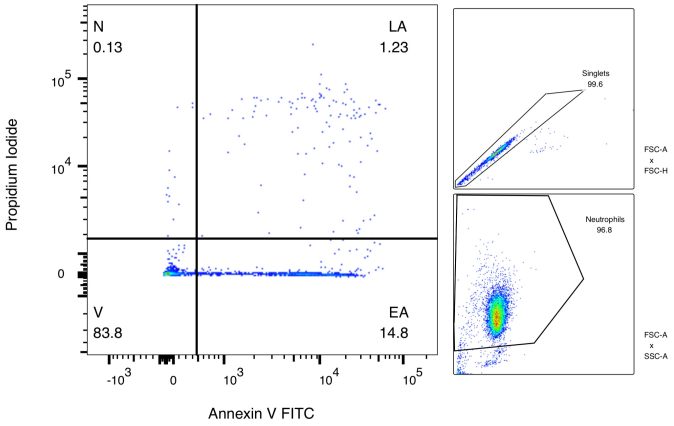

LPS

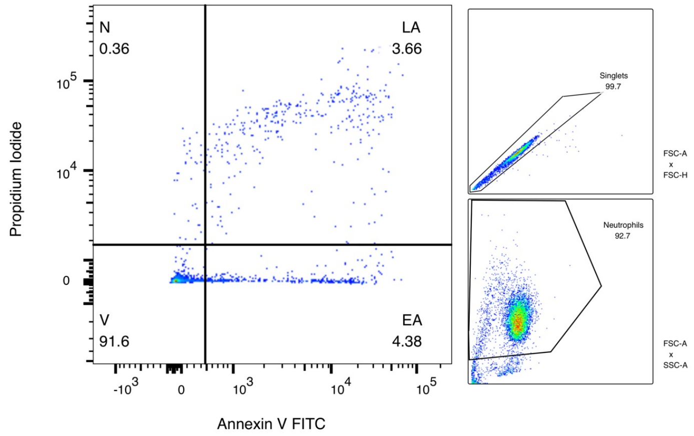

LPS+1,25D

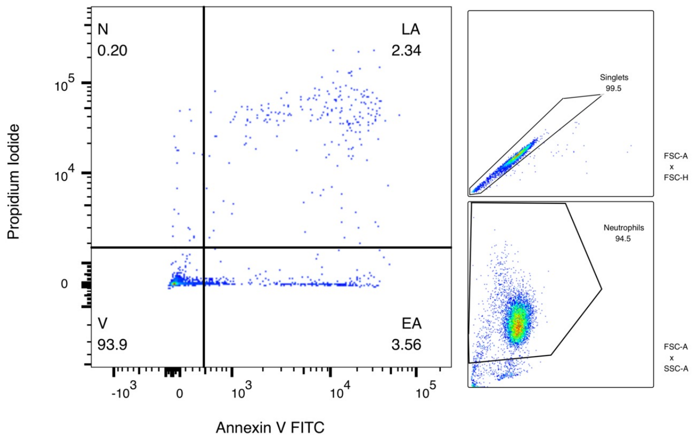

Fig. S1. Neutrophil viability after 6h as assessed by Annexin V/Propidium Iodide staining. Representative of 3 biological replicates.

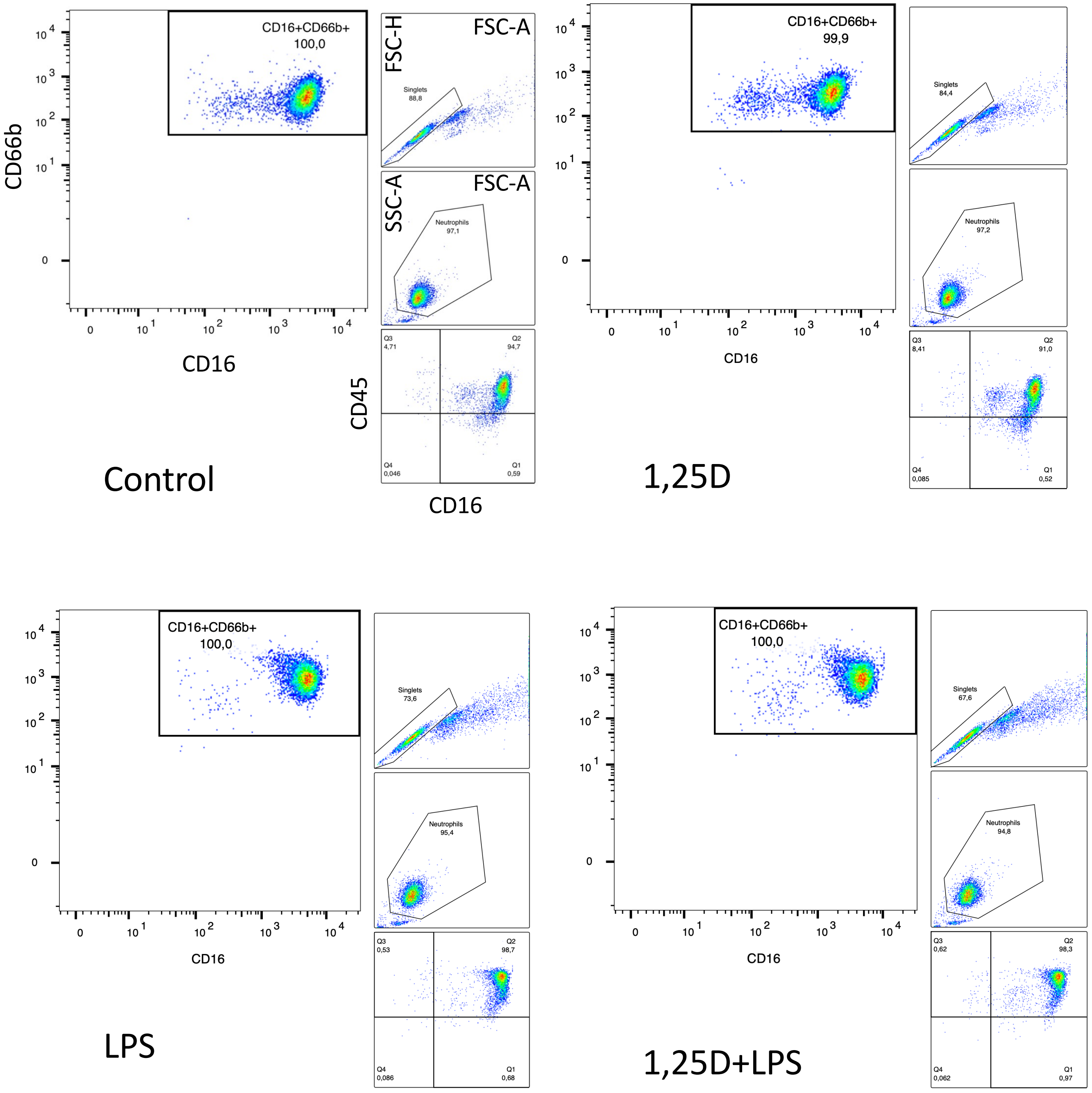

Fig. S2. Neutrophil purity after 6h as assessed by CD45, CD16 and CD66b positivity. Representative of 3 biological replicates.

		Counts per gene				
Condition	Donor	*VDR*	*CYP27B1*	*MAFF*	*BACH1*	Average
C	1	26241	6	24428	104186	4540
	2	25517	35	23384	84611	4319
	3	29417	18	14643	109295	4592
D	1	26981	9	17580	91644	4532
	2	27926	51	19239	66368	4461
	3	24376	11	12870	79464	4677
LPS	1	27700	9	59213	100157	4652
	2	23756	114	69525	76424	4411
	3	19686	22	129788	145788	4493
LPS+D	1	27028	13	52652	145389	5464
	2	18095	73	55700	95992	4245
	3	15668	13	104927	132749	4748

Table S1. Gene counts of *VDR, CYP27B1, MAFF* and *BACH1*, as well as average gene counts based on neutrophil RNAseq for each donor of C, 1,25D (D), LPS, and LPS+D-treated neutrophils.

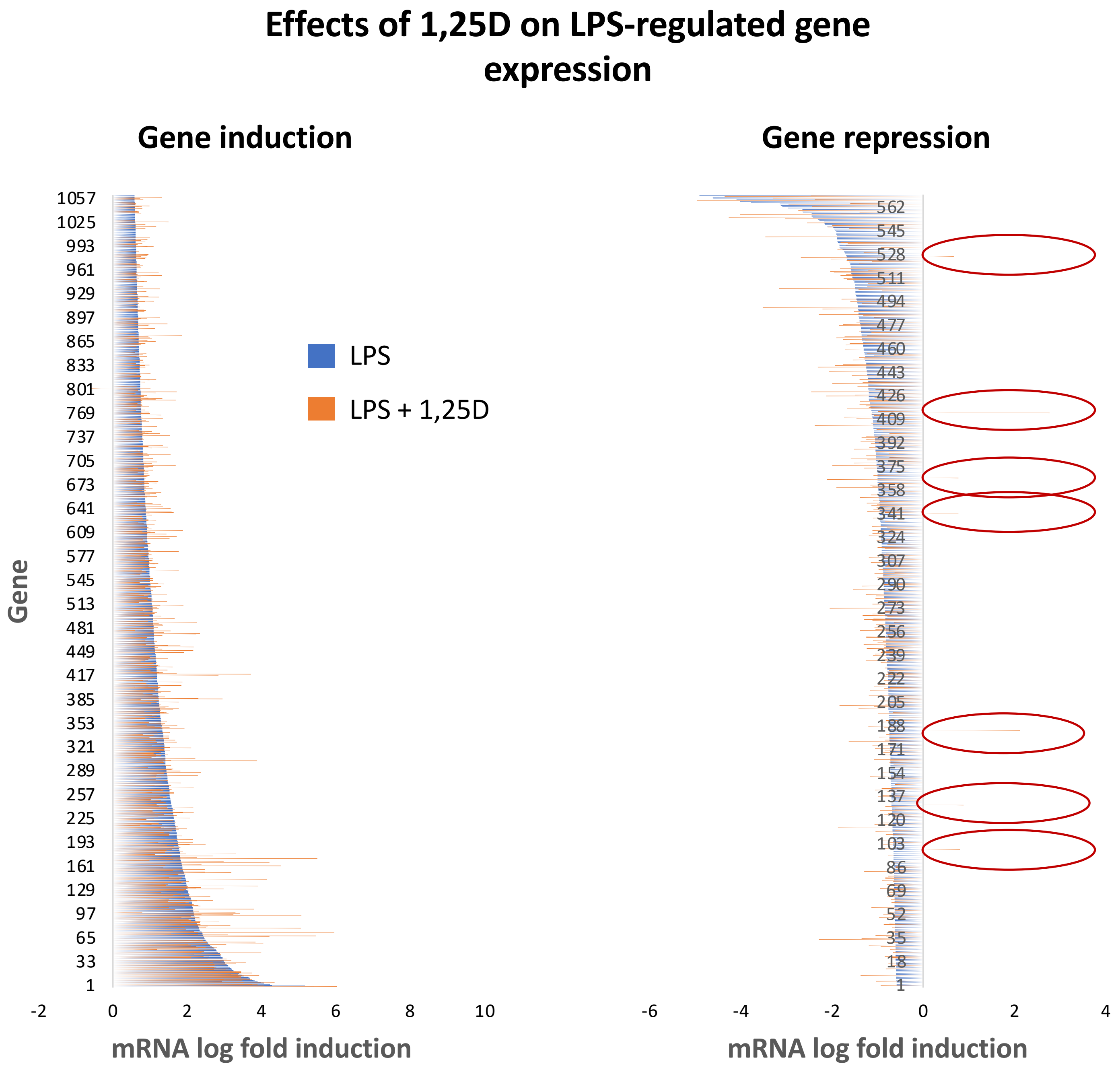

Fig. S3. Effects of 1,25D on LPS-regulated gene expression based on neutrophil RNAseq. Encircled in red are instances of LPS+1,25D-mediated repression on LPS-induced gene expression.

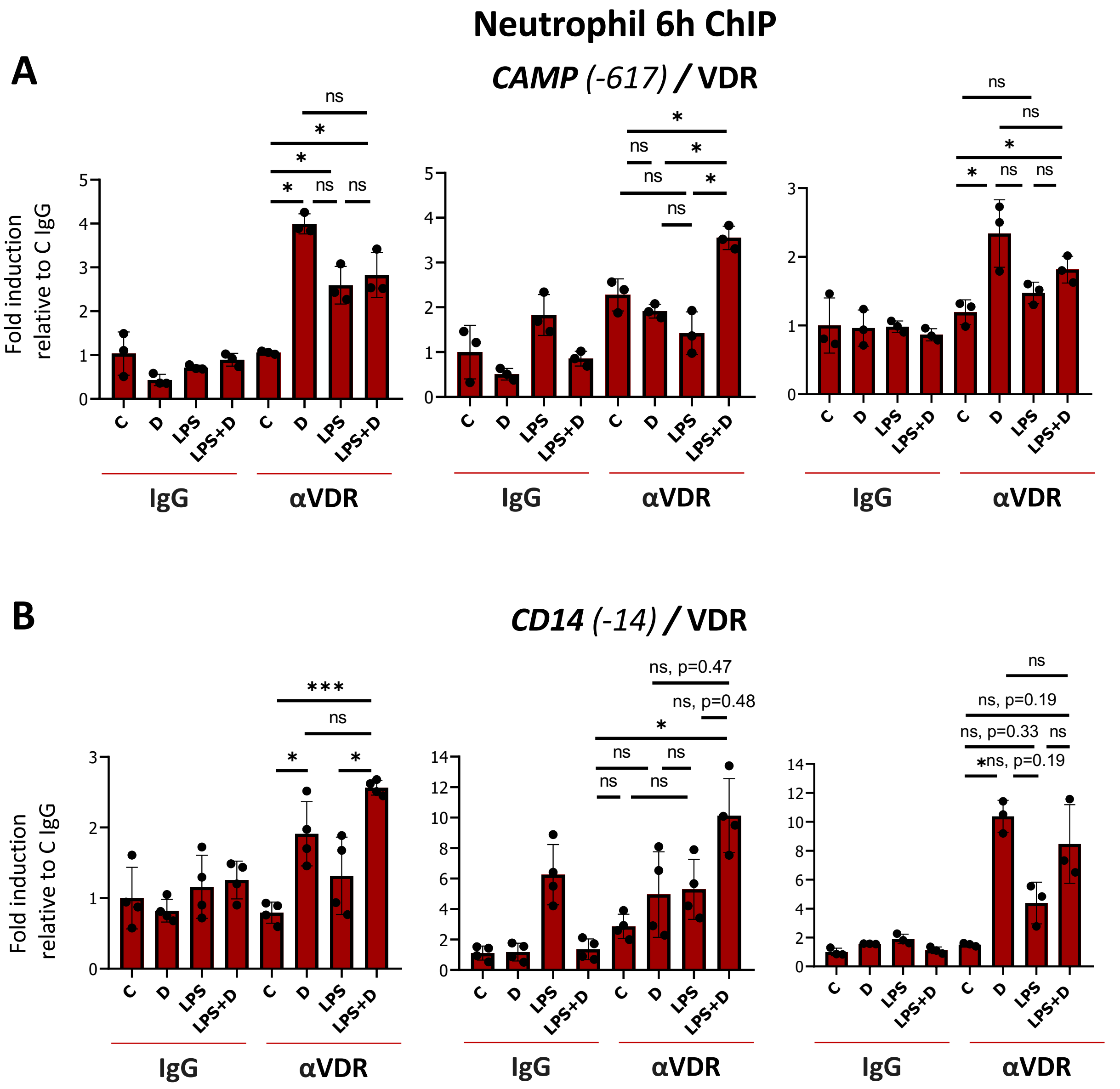

Fig. S4. Analysis of the association of VDR with regulatory regions of **(A)** *CAMP* and **(B)** *CD14* by ChIP assay in individual isolates of control-, 1,25D-, LPS- or LPS+1,25D- treated neutrophils for 6h (not including the representative data). Graphics are mean ± SD from at least 3 technical replicates from a biological sample and paired one-way ANOVAs followed by Tukey's *post hoc* test for multiple comparisons were used (*P ≤ 0.05, **P ≤ 0.01, ***P ≤ 0.001, and ns ≥0.05). ChIP values are normalized to input for each condition and expressed as a fold enrichment relative to IgG control.

Neutrophil 6h ChIP

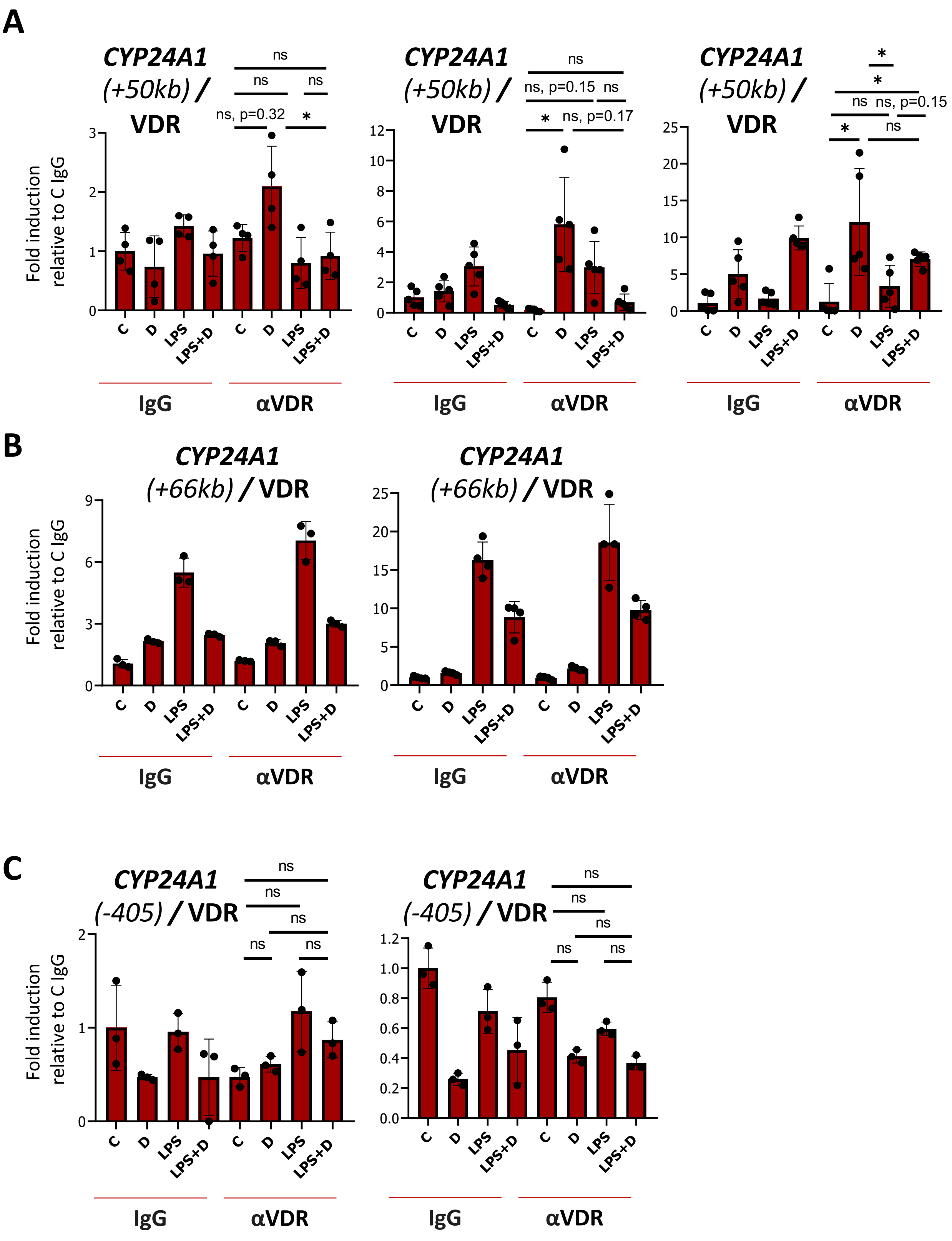

Fig. S5. Analysis of the association of VDR with regulatory regions of *CYP24A1* by ChIP assay in individual isolates of control-, 1,25D-, LPS- or LPS+1,25D- treated neutrophils for 6h (not including the representative data). Graphics are mean ± SD from at least 3 technical replicates from a biological sample and paired one-way ANOVAs followed by Tukey's *post hoc* test for multiple comparisons were used (*P ≤ 0.05 and ns ≥0.05). ChIP values are normalized to input for each condition and expressed as a fold enrichment relative to IgG control.

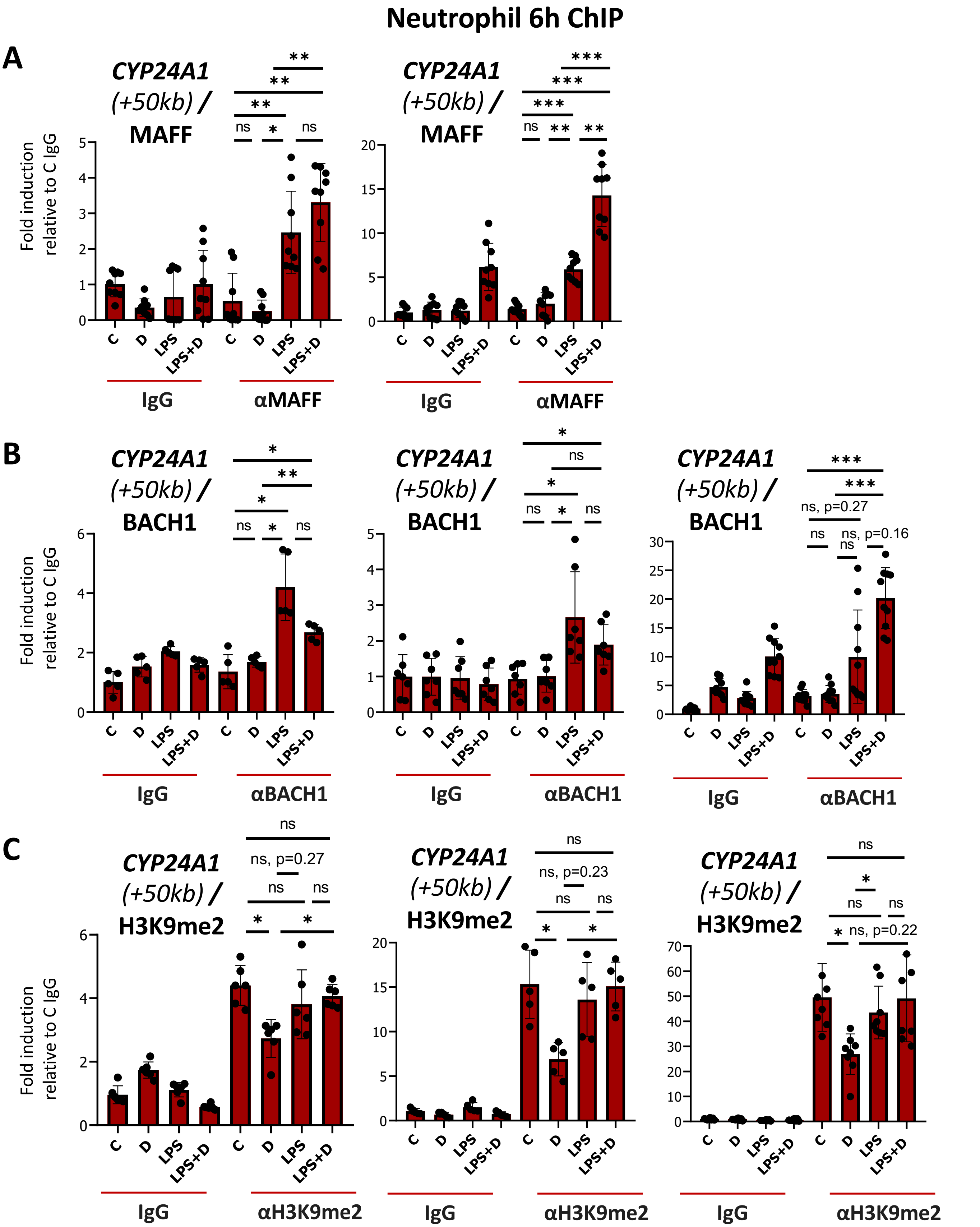

Fig. S6. Analysis of the association of **(A)** MAFF, **(B)** BACH1, and **(C)** H3K9me2 with regulatory regions of *CYP24A1* by ChIP assay in individual isolates of control-, 1,25D-, LPS- or LPS+1,25D- treated neutrophils for 6h (not including the representative data). Graphics are mean ± SD from at least 4 technical replicates from a biological sample and paired one-way ANOVAs followed by Tukey's *post hoc* test for multiple comparisons were used (*P ≤ 0.05, **P ≤ 0.01, ***P ≤ 0.001, and ns ≥0.05). ChIP values are normalized to input for each condition and expressed as a fold enrichment relative to control IgG.

GEO/ ENCODE accession	Target	Platform	Assembly	Reference	Cell Type	Treatment
GSE31939	VDR	Illumina Genome Analyzer IIx	hg18	Meyer et al	Colonic	3h 100 nM 1,25D
GSE124576	VDR	Illumina HiSeq 2500	hg19	Baumann et al	Primary prostate epithelial	2h 50nM 1,25D
GSE230578	VDR	Illumina HiSeq 4000	hg19	Oda et al	Keratinocytes	4h 100 nM 1,25D
GSE89431	VDR	Illumina HiSeq 2000	hg19	Seuter et al	THP-1	2h 100 nM 1,25D<br>24h 100 nM 1,25D
GSE27438	VDR	Illumina Genome Analyzer II	hg19	Heikkinen et al	THP-1	40 min 10 nM 1,25D
GSE51181	VDR	Illumina Genome Analyzer II	hg19	Carlberg et al	PMA-differentiated THP-1	60 min 100 nM 1,25D<br>120 min 100 nM 1,25D
ENCSR000EEC	MAFF	Illumina Genome Analyzer	Hg38, hg19	Michael Snyder, Stanford	HepG2	
ENCSR000EBQ	BACH1	Illumina Genome Analyzer	Hg38, hg19	Michael Snyder, Stanford	human H1-embryonic stem cells	

Table S2. ChIPseq datasets included in the study. All datasets included in the study with associated GEO or ENCODE accession number, target, platform, assembly, lab or publication reference, treatment and cell type.

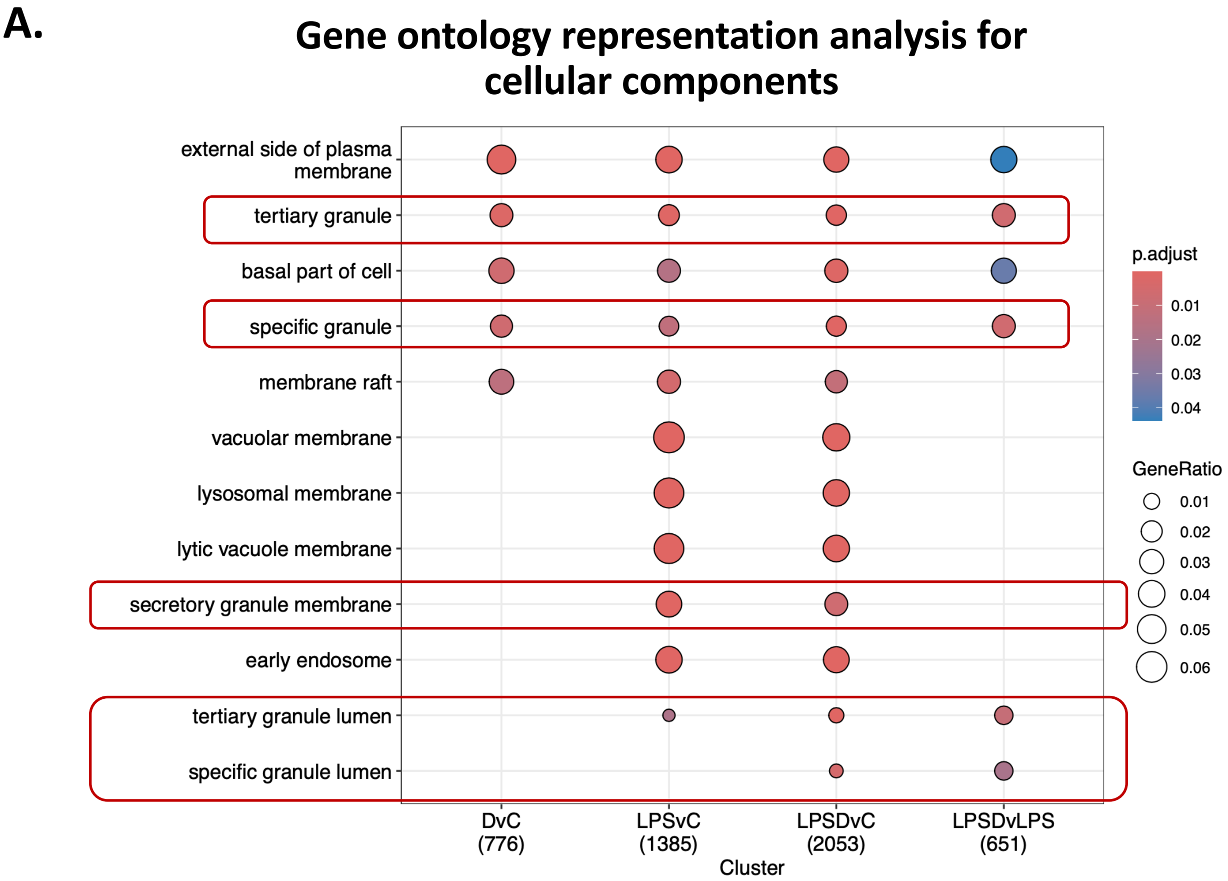

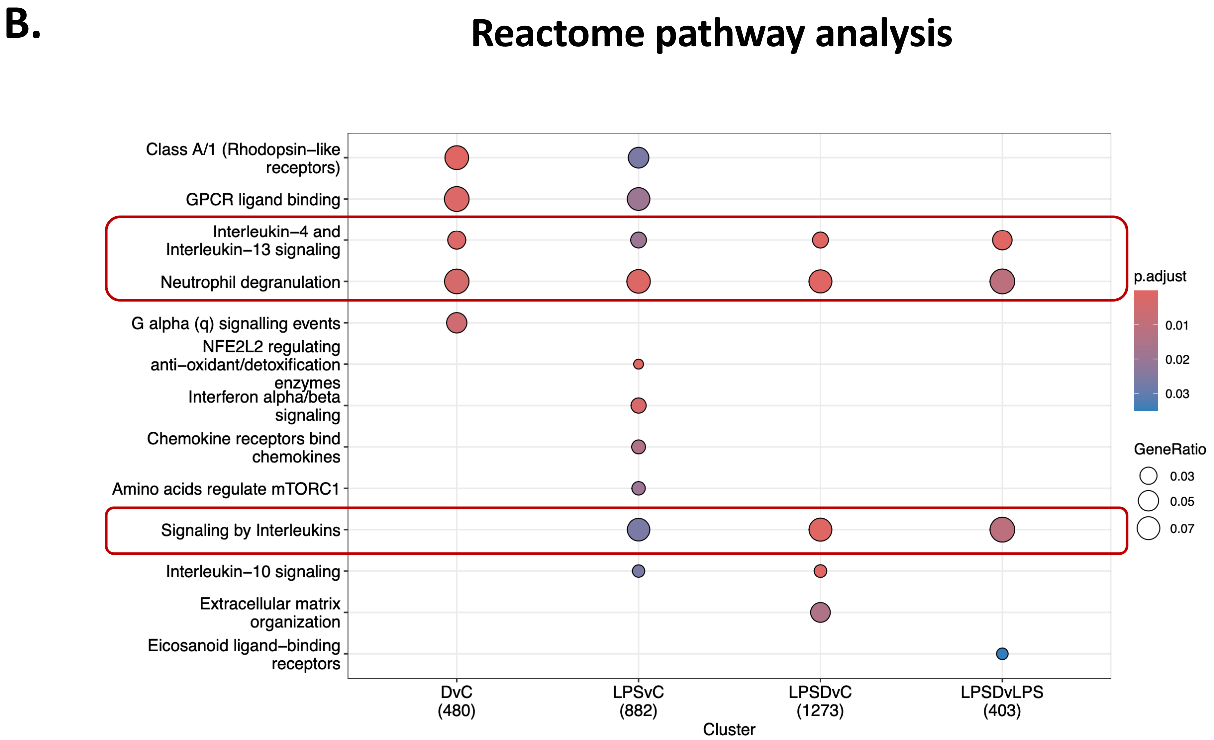

Fig. S7. Enriched gene ontology representation analysis for cellular components **(A)** and reactome pathway analyses **(B)** of 1.5-fold regulated genes from neutrophil RNAseq. DvC, LPSvC, LPSDvC = 1,25D, LPS, and LPS+1,25D-treated relative to control cells, respectively. LPSDvLPS shows processes enriched from genes in LPS+1,25D-treated cells relative to LPS-stimulated neutrophils.

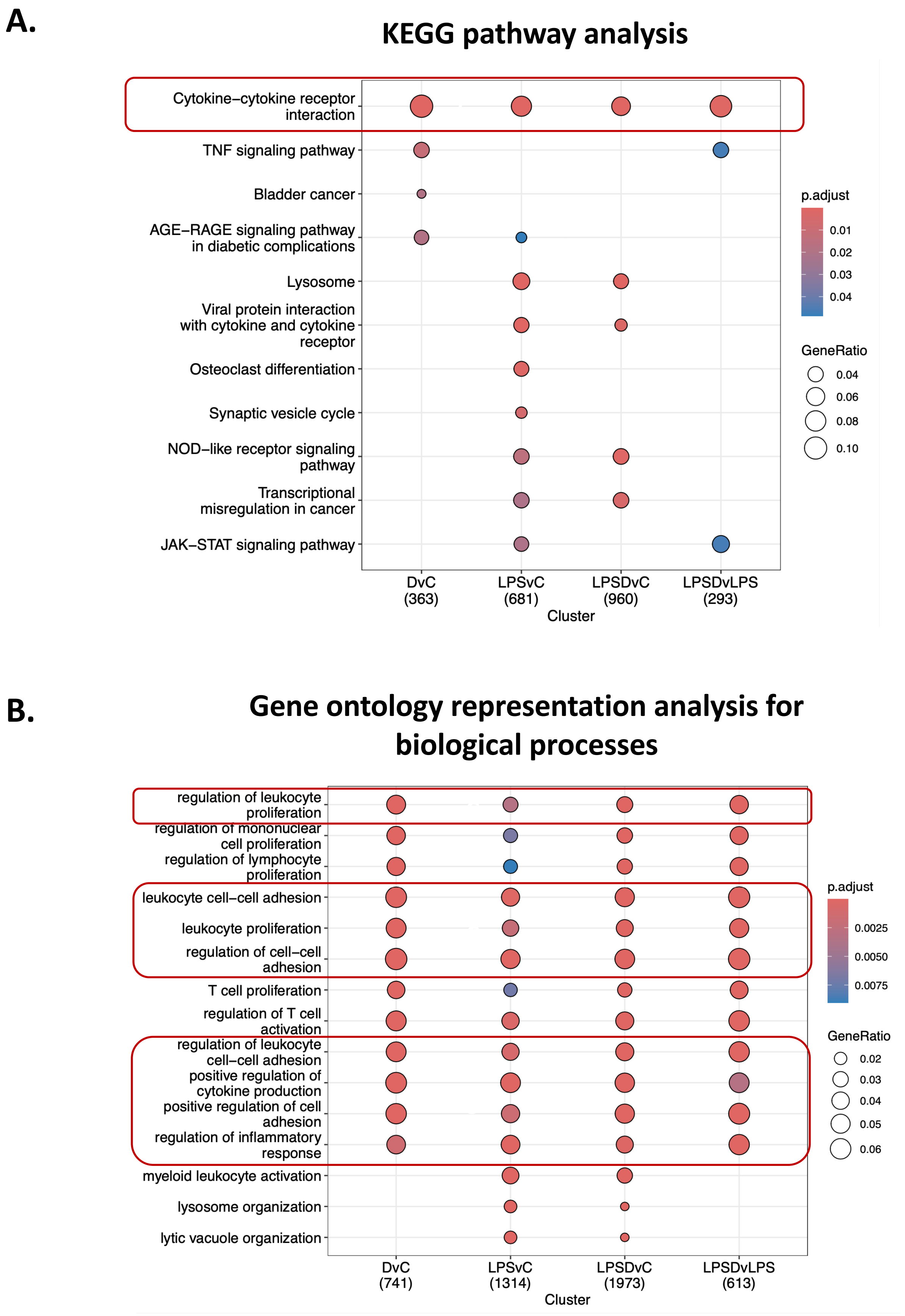

Fig. S8. Enriched KEGG pathways **(A)** and gene ontology representation analysis for biological processes **(B)** of 1.5-fold regulated genes from neutrophil RNAseq. DvC, LPSvC, LPSDvC = 1,25D, LPS, and LPS+1,25D-treated relative to control cells, respectively. LPSDvLPS shows processes enriched from genes in LPS+1,25D-treated cells relative to LPS-stimulated neutrophils.

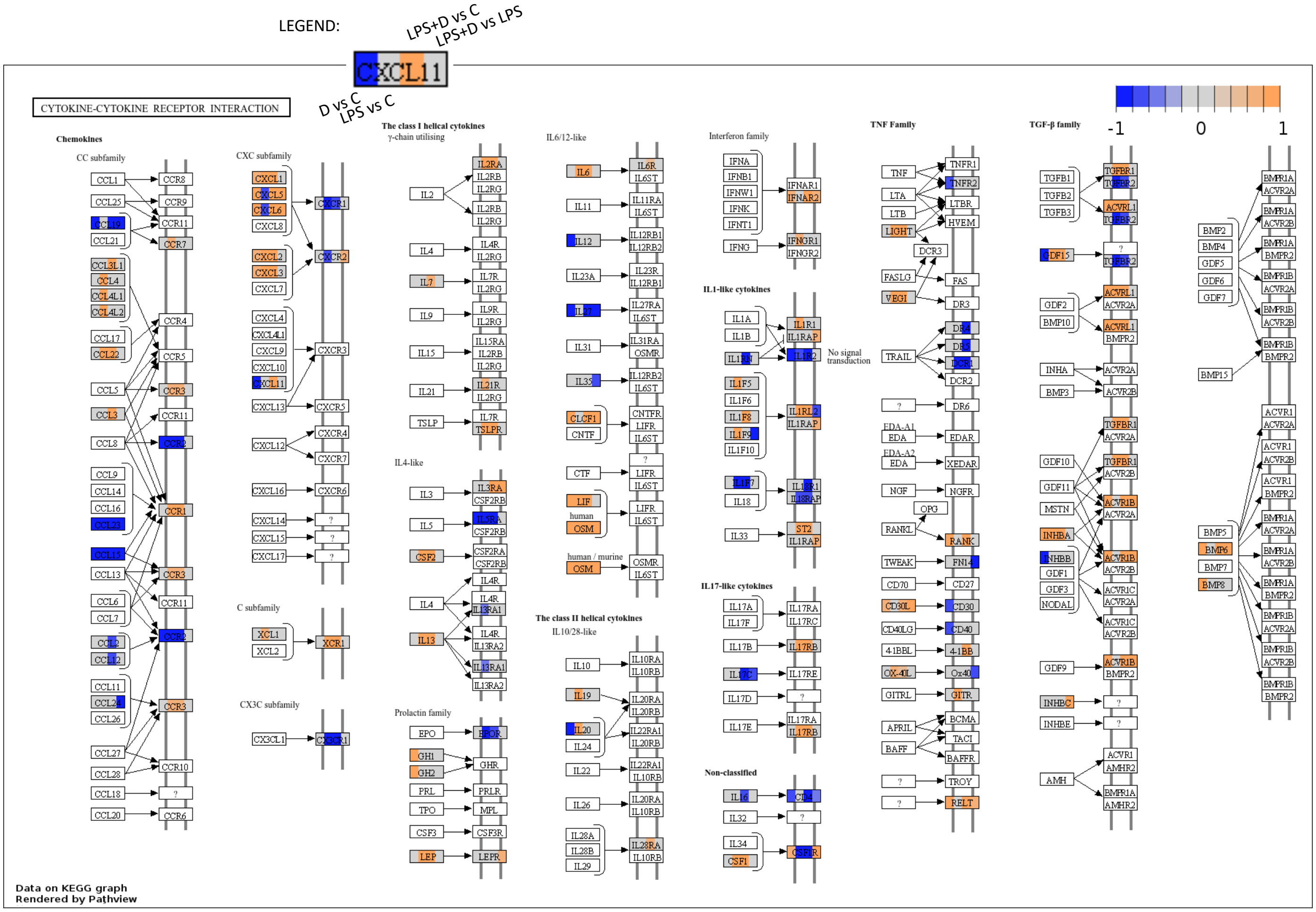

Fig. S9. Enriched cytokine-cytokine receptor interaction (KEGG pathway) of 1.5-fold (log fold change of 0.58) regulated genes from 1,25D-, LPS- and LPS+1,25D-treated neutrophils relative to control, as well as LPS+1,25D-stimulated cells vs. LPS-treated. Scale is from log fold change relative to C of -1 (dark blue) to +1 (orange).

	Type of granule							
Gene	Azurophil (1°)	Specific (2°)	Tertiary (3°)	Secretory vesicle	Host defense	Anti-inflammatory	Cytoskeleton organization	Reference(s)
CAMP	✓	✓	✓		✓			Bucki et al, White
*DEFA1**	✓	✓			✓			Ganz
CRISPLD2				✓	✓	✓		Gibbs et al, Trexler et al, Zhang et al
LRG1		✓	✓		✓			Ng and Xavier, Camilli et al
ALOX5				✓	✓			Caffrey-Carr et al
*CDA**			✓		✓			Mok et al
ORM2		✓				✓		Jo et al, Lee et al
*ORM1**		✓	✓			✓		Hochepied et al
*CD93**		✓	✓		✓			Greenlee et al, Jeon et al
GGH		✓	✓	✓	✓			Fernández-Villa et al
CTSZ		✓			✓	✓		Anes et al, Bangalore et al
DBNL				✓			✓	Ghosh et al
DNAJC3								
GM2A	✓							
*CR1**				✓	✓			Vandendriessche et al
*MMP8***			✓			✓***		Gutierrez-Fernandez et a, Owen et al, Gonzalez-Lopez et al, Manicone and McGuire

* D vs C only

** LPS+D vs LPS only

***MMP8 may be anti- (Gutierrez-Fernandez et a, Owen et al, Gonzalez-Lopez et al, Manicone and McGuire) or pro-inflammatory (Lauhio et al, Solan et al, Vandenbroucke et al) under different contexts.

Table S3. Genes within the degranulation network regulated by 1,25D, in the presence and absence of LPS, (red rectangle from Fig 5A) that encode secreted proteins classified into their respective granule compartment(s). Genes classified based on previous study on proteome profiling of human neutrophil granules.

References for Table S2:

Bucki R, Leszczyńska K, Namiot A, Sokołowski W. Cathelicidin LL-37: a multitask antimicrobial peptide. Archivum immunologiae et therapiae experimentalis. 2010 Feb;58:15-25.

White JH. Emerging roles of vitamin D-induced antimicrobial peptides in antiviral innate immunity. Nutrients. 2022;14(2):284.

Ganz T. Defensins: antimicrobial peptides of innate immunity. Nature reviews immunology. 2003 Sep 1;3(9):710-20.

Gibbs GM, Roelants K, O'bryan MK. The CAP superfamily: cysteine-rich secretory proteins, antigen 5, and pathogenesis-related 1 proteins—roles in reproduction, cancer, and immune defense. Endocrine reviews. 2008 Dec 1;29(7):865-97.

Trexler M, Bányai L, Patthy L. The LCCL module. European Journal of Biochemistry. 2000 Sep;267(18):5751-7.

Zhang H, Kho AT, Wu Q, Halayko AJ, Limbert Rempel K, Chase RP, Sweezey NB, Weiss ST, Kaplan F. CRISPLD2 (LGL1) inhibits proinflammatory mediators in human fetal, adult, and COPD lung fibroblasts and epithelial cells. Physiological Reports. 2016 Sep;4(17):e12942.

Ng A, Xavier RJ. Leucine-rich repeat (LRR) proteins: integrators of pattern recognition and signaling in immunity. Autophagy. 2011 Sep 1;7(9):1082-4.

Camilli, C., Hoeh, A.E., De Rossi, G., Moss, S.E. and Greenwood, J., 2022. LRG1: an emerging player in disease pathogenesis. Journal of biomedical science, 29(1), p.6.

Caffrey-Carr AK, Hilmer KM, Kowalski CH, Shepardson KM, Temple RM, Cramer RA, Obar JJ. Host-derived leukotriene B4 is critical for resistance against invasive pulmonary aspergillosis. Frontiers in immunology. 2018 Jan 11;8:1984.

Mok BY, de Moraes MH, Zeng J, Bosch DE, Kotrys AV, Raguram A, Hsu F, Radey MC, Peterson SB, Mootha VK, Mougous JD. A bacterial cytidine deaminase toxin enables CRISPR-free mitochondrial base editing. Nature. 2020 Jul 23;583(7817):631-7.

Jo M, Kim JH, Song GJ, Seo M, Hwang EM, Suk K. Astrocytic orosomucoid-2 modulates microglial activation and neuroinflammation. Journal of Neuroscience. 2017 Mar 15;37(11):2878-94.

Lee YS, Choi JW, Hwang I, Lee JW, Lee JH, Kim AY, Huh JY, Koh YJ, Koh GY, Son HJ, Masuzaki H. Adipocytokine orosomucoid integrates inflammatory and metabolic signals to preserve energy homeostasis by resolving immoderate inflammation. Journal of Biological Chemistry. 2010 Jul 16;285(29):22174-85.

Hochepied, T., Berger, F.G., Baumann, H. and Libert, C., 2003. α1-Acid glycoprotein: an acute phase protein with inflammatory and immunomodulating properties. Cytokine & growth factor reviews, 14(1), pp.25-34.

Greenlee MC, Sullivan SA, Bohlson SS. Detection and characterization of soluble CD93 released during inflammation. Inflammation research. 2009 Dec;58:909-19.

Jeon JW, Jung JG, Shin EC, Choi HI, Kim HY, Cho ML, Kim SW, Jang YS, Sohn MH, Moon JH, Cho YH. Soluble CD93 induces differentiation of monocytes and enhances TLR responses. The Journal of Immunology. 2010 Oct 15;185(8):4921-7.

Fernández-Villa D, Aguilar MR, Rojo L. Folic acid antagonists: antimicrobial and immunomodulating mechanisms and applications. International journal of molecular sciences. 2019 Oct 9;20(20):4996.

Anes E, Pires D, Mandal M, Azevedo-Pereira JM. Spatial localization of cathepsins: Implications in immune activation and resolution during infections. Frontiers in Immunology. 2022 Aug 3;13:955407.

Bangalore N, Travis J, Onunka VC, Pohl J, Shafer WM. Identification of the primary antimicrobial domains in human neutrophil cathepsin G. Journal of Biological Chemistry. 1990 Aug 15;265(23):13584-8.

Ghosh A, Enderlein J, Butkevich E. Dimerization of human drebrin-like protein governs its biological activity. Biochemistry. 2020 Apr 13;59(16):1553-8.

Vandendriessche S, Cambier S, Proost P, Marques PE. Complement receptors and their role in leukocyte recruitment and phagocytosis. Frontiers in Cell and Developmental Biology. 2021 Feb 11;9:624025.

Gutiérrez-Fernández A, Inada M, Balbín M, Fueyo A, Pitiot AS, Astudillo A, Hirose K, Hirata M, Shapiro SD, Noël A, Werb Z. Increased inflammation delays wound healing in mice deficient in collagenase-2 (MMP-8). The FASEB journal: official publication of the Federation of American Societies for Experimental Biology. 2007 Aug;21(10):2580.

Owen CA, Hu Z, Lopez-Otin C, Shapiro SD. Membrane-bound matrix metalloproteinase-8 on activated polymorphonuclear cells is a potent, tissue inhibitor of metalloproteinase-resistant collagenase and serpinase. The Journal of Immunology. 2004 Jun 15;172(12):7791-803.

Manicone AM, McGuire JK. Matrix metalloproteinases as modulators of inflammation. InSeminars in cell & developmental biology 2008 Feb 1 (Vol. 19, No. 1, pp. 34-41). Academic Press.

Gonzalez-Lopez A, Aguirre A, Lopez-Alonso I, Amado L, Astudillo A, Fernández-García MS, Suarez MF, Batalla-Solis E, Colado E, Albaiceta GM. MMP-8 deficiency increases TLR/RAGE ligands S100A8 and S100A9 and exacerbates lung inflammation during endotoxemia. PLoS One. 2012 Jun 29;7(6):e39940.

Lauhio A, Hästbacka J, Pettilä V, Tervahartiala T, Karlsson S, Varpula T, Varpula M, Ruokonen E, Sorsa T, Kolho E. Serum MMP-8,-9 and TIMP-1 in sepsis: high serum levels of MMP-8 and TIMP-1 are associated with fatal outcome in a multicentre, prospective cohort study. Hypothetical impact of tetracyclines. Pharmacological research. 2011 Dec 1;64(6):590-4.

Solan PD, Dunsmore KE, Denenberg AG, Odoms K, Zingarelli B, Wong HR. A novel role for matrix metalloproteinase-8 in sepsis. Critical care medicine. 2012 Feb 1;40(2):379-87.

Vandenbroucke RE, Dejonckheere E, Van Lint P, Demeestere D, Van Wonterghem E, Vanlaere I, Puimege L, Van Hauwermeiren F, De Rycke R, Mc Guire C, Campestre C. Matrix metalloprotease 8-dependent extracellular matrix cleavage at the blood–CSF barrier contributes to lethality during systemic inflammatory diseases. Journal of Neuroscience. 2012 Jul 18;32(29):9805-16.

Targeted gene	Forward sequence	Reverse sequence
**Primers RT/qPCR**		
ALOX5	5'-ACTTCGCCGACTTTGAGAAA-3'	5'-GTGCAGGGGTCTGTTTTGTT-3'
BACH1	5'-GCTGGATTTAGCGAAGACTGG-3'	5'-CTTCCGCTGGTCATTAAGGC-3'
CAMP	5’-GACAGTGACCCTCAACCAGG-3’	5’-AGGGCACACACTAGGACTCT-3’
CD14	5'-GAACCTTGTGAGCTGGACGA-3'	5'-GCATCGACGCGCTTTAGAAA-3'
CDA	5'-TGCCCCTACAGTCACTTTCC-3'	5'-TCTCTCATGACTTGCCTGCA-3'
CTSZ	5’-AGGCCATGTCACTAGAAGCG-3'	5'-GCTGTCCTCGCCATCCAATA-3'
CXCL6	5'-AAGTTTGTCTGGACCCGGAA-3'	5'-AACTTCAGGGAGAAGCGTAGG-3'
CYP24A1	5'-CCACGTTGAAGACTTGTACAGC-3'	5'-GAGAAGCCCAAATCTCTTCTCA-3'
CYP27B1	5'-CCTGACCCACTTCCTGTTCC-3'	5'-CTCCCGCCAATAGCAACTCT-3'
DEFA1	5'-TTGCATGGGACGAAAGCTTG-3'	5'-TAGCGACGTTCTCCTGCAAT-3'
G0S2	5'-CTAAGGTCATTCCCGCCTCC-3'	5'-AGCACGTACAGCTTCACCAT-3'
HBEGF	5'-ATCTTCCGTGGTCAGAGTGC-3'	5'-GGGACCAGGAAAGCTACAGG-3'
IL36G	5’-ATCACATGCAAGTATCCAGAGG-3’	5’-ACGGTAGAAAAGGAAGGGTTTC-3’
ITGAM	5'-AGAACAACATGCCCAGAACC-3'	5'-GCGGTCCCATATGACAGTCT-3'
ITGAX	5'-AACATGTACCTCACCGGACTCT-3'	5'-TCTCTGGAACTGGCTTATCACA-3'
LRG1	5'-GATCTAACCCGAAACGCCCT-3'	5'-GGTTCTCCCCAAGGTCAAGG-3'
MAFF	5’-ATCCCCTATCCAGCAAAGCTC-3’	5’-TTGAGCCGTGTCACCTCCTC-3’
ORM1	5'-AACACCACCTACCTGAATGTCC-3'	5'-GAGGATCAGCAAGTGAGCGA-3'
OSM	5'-CTCCAGAAGCAGACAGATCTCA-3'	5'-TGCAGTGCTCTCTCAGTTTAGG-3'
SERPINB1	5'-TGGCTACATCGAGGACCTTAAG-3'	5'-AGGTCGGAGTTGAGAGTGTAAC-3'
SLC2A3	5'-GTGTGCTTTAGCTTGAAAAGGTG-3'	5'-GCCTTACTGCCAACCTACTGT-3'
VDR	5'-GCCCACCATAAGACCTACGA-3'	5'-CCATCATTCACACGAACTGG-3'
ZC2HC1C	5’-GAAGTCGTGCAGTACAGGTGAG-3’	5’-GCAAGAACAGTACCAACCACAG-3’
**Primers ChIP-qPCR**		
CAMP-617	5'-GGGATAGATGGAGCAGAGCC-3'	5'-AAGACAGTAGCCACCCCCAA-3'
CD14-14	5'-CCAAGACCCTACACTCACCA-3'	5'-GCAGCCGAAGAGTTCACAAG-3'
CYP24A1+50	5'-AGGCAAGACTTTGCATTC-3'	5'-TTTCTCAGGCTCACCAAACC-3'
CYP24A1+66	5'-CTTGTTTTCTGCTGGGTTCA-3'	5'-AGGATGCCGTTTGTTCTCAC-3'
CYP24A1-405	5'-GCTATGTTCGCTGGGCG-3'	5'-GCATGACTTCCTGGGGGTTA-3'

Table S4. Primer sequences for RT/qPCR and ChIP-qPCR.
